# Comprehensive Expression Profiling of Rice Tetraspanin Genes Reveals Diverse Roles During Development and Abiotic Stress

**DOI:** 10.3389/fpls.2015.01088

**Published:** 2015-12-11

**Authors:** Balaji Mani, Manu Agarwal, Surekha Katiyar-Agarwal

**Affiliations:** ^1^Department of Plant Molecular Biology, University of Delhi South CampusNew Delhi, India; ^2^Department of Botany, University of DelhiDelhi, India

**Keywords:** rice, tetraspanin, abiotic stress, hormone, nutrient deprivation, gene expression

## Abstract

Tetraspanin family is comprised of evolutionarily conserved integral membrane proteins. The incredible ability of tetraspanins to form ‘micro domain complexes’ and their preferential targeting to membranes emphasizes their active association with signal recognition and communication with neighboring cells, thus acting as key modulators of signaling cascades. In animals, tetraspanins are associated with multitude of cellular processes. Unlike animals, the biological relevance of tetraspanins in plants has not been well investigated. In *Arabidopsis* tetraspanins are known to contribute in important plant development processes such as leaf morphogenesis, root, and floral organ formation. In the present study we investigated the genomic organization, chromosomal distribution, phylogeny and domain structure of 15 rice tetraspanin proteins (OsTETs). OsTET proteins had similar domain structure and signature ‘GCCK/R’ motif as reported in *Arabidopsis*. Comprehensive expression profiling of *OsTET* genes suggested their possible involvement during rice development. While *OsTET9* and *10* accumulated predominantly in flowers, *OsTET5, 8*, and *12* were preferentially expressed in root tissues. Noticeably, seven *OsTETs* exhibited more than twofold up regulation at early stages of flag leaf senescence in rice. Furthermore, several *OsTETs* were differentially regulated in rice seedlings exposed to abiotic stresses, exogenous treatment of hormones and nutrient deprivation. Transient subcellular localization studies of eight OsTET proteins in tobacco epidermal cells showed that these proteins localized in plasma membrane. The present study provides valuable insights into the possible roles of tetraspanins in regulating development and defining response to abiotic stresses in rice. Targeted proteomic studies would be useful in identification of their interacting partners under different conditions and ultimately their biological function in plants.

## Introduction

Tetraspanins belong to a superfamily of highly evolutionarily conserved integral membrane proteins with typical arrangement of four transmembrane (TM) domains (TM1-4), two extracellular loops (EC1 and EC2) of unequal sizes, small intracellular loop (IL), short N-, C-terminal cytoplasmic tails and a signature motif (‘GCCK/RP’ in plants and ‘CCG’ in animals) in EC2 (Huang et al., 2005; Boavida et al., 2013). They are conspicuously present in all multicellular organisms and unicellular protozoan amoeba, but are remarkably absent in yeast. Till date, 33 tetraspanins in humans, 37 in *Drosophila melanogaster*, 20 in C*aenorhabditis elegans* (Huang et al., 2005), 4 in fungi (Gourgues et al., 2002), and 17 in *Arabidopsis thaliana* (Garcia-Espana et al., 2008) have been reported. Majority of the tetraspanin proteins are targeted to plasma membrane (PM) where they are believed to recognize extracellular signals, which may cause conformational changes in their cytoplasmic domain resulting in the activation of specific signaling cascades (Levy and Shoham, 2005; Andreu and Yanez-Mo, 2014). The remarkable ability of tetraspanins to form multi-molecular complexes with each other (secondary interactions) and other partner proteins (primary interactions) enables them to form ‘tetraspanin-enriched microdomain’ (TEM) or ‘tetraspanin web’. They are believed to act as ‘molecular organizers or facilitators’ and they actively participate in the coordination of intracellular signaling pathways with cytoskeleton as they interact with various proteins, including integrins, immunoglobulin superfamily, major histocompatibility complex, growth factor receptors, signaling molecules and receptor proteins (Hemler, 2005). In animals tetraspanins are crucial in regulating cell adhesion, proliferation, motility, cell-to-cell interaction, fusion, and intracellular trafficking (Yunta and Lazo, 2003; Hemler, 2005; Berditchevski and Odintsova, 2007). Functionally tetraspanin proteins are associated with multitude of physiological processes such as pathogenesis, fertilization, induction of immune responses, and tumor progression and suppression (Hemler, 2005; Zoller, 2009).

The dynamic nature of tetraspanins to interact with multiple molecules and their diverse biological roles enthused plant biologists to look into the diversity in structure and function of plant tetraspanins. However, plant tetraspanins have not been investigated in much details and very little information is available on the function of these proteins in plants. Survey of several plant genomes for tetraspanin proteins revealed that they are ubiquitously found in multicellular plant species, but not in unicellular plant forms (Van Bel et al., 2012; Wang et al., 2012). Owing to redundancy in their function, mutations for loss-of-function phenotypes have generated limited information on the specific function of tetraspanin proteins in plants. The first evidence for the involvement of tetraspanin protein in plant development was provided by studies on *ekeko* mutant harboring a T-DNA insertion in *Arabidopsis TET1* gene (Olmos et al., 2003). Severe developmental defects in leaf patterning, root growth and floral organ formation, probably due to dysregulated cell differentiation, suggested their role in regulating key developmental processes in plants (Olmos et al., 2003). Another mutant allele of *TET1*, which was named as *TORNADO2 (trn2)*, exhibited defects in early leaf development (Cnops et al., 2006). A study by Chiu et al. (2007) on *trn2* mutant implicated tetraspanin in defining cellular decisions at the periphery of shoot apical meristem (SAM). It was found that *TRN2* could partially compensate for the loss of shoot meristemless gene, *STM*, which contributes to establishment and maintenance of SAM in *Arabidopsis*. Lieber et al. (2011) demonstrated that *Arabidopsis WINDHOSE* (*WIN*) gene that encodes for small GYPP-repeat proteins functions with *TRN2* in promoting megasporogenesis. However, the molecular role of *TRN2* in regulating phase transition from somatic to reproductive cell fate needs to be explored. Based on the expression studies in different cell types in reproductive tissues, Boavida et al. (2013) proposed involvement of TET proteins in reproductive development of *Arabidopsis*. Split ubiquitin assays in yeast showed that several members of *Arabidopsis* tetraspanin family associate strongly as homomers or heteromers, which could explain the functional redundancy due to dynamicity and diversity in the molecular interactions among these proteins. Recently Wang et al. (2015) performed a more detailed study advocating the role of tetraspanin in different aspects of *Arabidopsis* development and further proposed transcription factor (TF)-TET regulatory network for prediction of molecular function of TETs in various plant pathways.

In the present study efforts were made to perform a genome-wide analysis of tetraspanin protein family in rice with respect to the identification of true tetraspanin protein encoding genes, their genomic organization, phylogenetic analysis and motif analysis of predicted tetraspanin proteins followed by identification of *cis*-regulatory elements in putative gene promoters. We generated expression pattern information of tetraspanin genes in vegetative and reproductive tissues of rice. Detailed expression profiling during progression of senescence of flag leaf showed that several of these genes were induced at early stage of senescence, pointing toward their role in regulating senescence in plants. Additionally, rice tetraspanin genes were regulated by abiotic stresses, nutrient deprivation, and exogenous application of phytohormones in rice seedlings indicating that these proteins may act versatile as signal relays and participate in mediating multitude of biological processes. We generated a comprehensive expression atlas of rice tetraspanin genes along with the information on their subcellular localization, all of which would be useful in understanding the biological function of this multifunctional protein family in plants.

## Materials and Methods

### Identification of Tetraspanin Gene Family Members in Rice

To identify members of rice tetraspanin (*OsTET*) gene family keyword search was performed using ‘TETRASPANIN’ in RGAP v7^1^ and Phytozome v10.3^2^ databases. The second strategy involved exploring the complete rice proteome available at RGAP pseudo molecules v7.0 for the identification of transmembrane (TM) containing proteins using TMHMM v2.0 (TM Helices Hidden Markov Model^3^). Further shortlisting was done for the proteins containing four TM proteins. Subsequently these proteins were screened for the presence of characteristic features of tetraspanin proteins: 4TM, 2ECL of unequal sizes, 1ICL, 9 cysteine residues in EC2, ‘GCCK’ motif, short N-, C-terminal tails. Perl scripts were designed for analyzing the output obtained at each step. The outputs obtained by two strategies were compared and members commonly present in both sets were identified as ‘true tetraspanin proteins’. All predicted protein sequences were subjected to SMART (Sequence Modular Architecture Research Tool), Pfam and InterPro analysis to confirm the presence of TM domains and tetraspanin-specific ‘GCCK’ motif. All the *in silico* analyses were carried out using available nucleotide or protein sequences of rice variety, Nipponbare.

### Nomenclature, Chromosomal Distribution, and Gene Duplication of Rice Tetraspanins

The *OsTET* genes were mapped on 12 chromosomes of rice by using chromosome map tool in Oryzabase database^4^ and a map of was drawn on the basis of output generated. Tetraspanins were sequentially numbered on the basis of their location on rice chromosomes. Gene duplication analyses were performed using PGDD (Plant Genome Duplication Database^5^). Segmental duplication of *OsTET* genes was determined with the maximal length distance allowed between colinear gene pairs of 500 kb using data available at RGAP^6^. Genes were considered as tandemly duplicated if they belong to the same family, located on the same chromosome and not separated by a maximum of 10 unrelated genes (Du et al., 2013; Jiang et al., 2013).

### Phylogenetic Analysis of Rice and *Arabidopsis* Tetraspanin Proteins

*Arabidopsis* (*A. thaliana*) tetraspanin protein sequences were obtained from The *Arabidopsis* Information Resource (TAIR^7^). All predicted full-length rice *(Oryza sativa)* tetraspanin protein sequences were downloaded from Rice Genome Annotation Project (RGAP) database. Multiple sequence alignment of these proteins was carried out with ClustalX 2.1. The sequence alignment was imported to Molecular Evolutionary Genetic Analysis (MEGA) v6.0 for generating an un-rooted neighbor-joining phylogenetic tree with 1000 bootstrap value. The proteins were clustered together in respective clades based on significant bootstrap value (≥50%).

### Analysis of Conserved Motifs in Rice Tetraspanin Proteins

To identify characteristic structural components and the divergence in tetraspanin proteins in rice, corresponding predicted protein sequences were aligned by ClustalX 2.1. TM domains were predicted by SMART (Sequence Modular Architecture Structure Tool^8^). Potential palmitoylation sites were predicted with Palmitoylation CSS-Palm 2.0 (Ren et al., 2008) and NetNGlyc 1.0 server^9^ was employed to identify potential *N*-glycosylation sites. OsTET proteins identity and similarity matrix was generated by using online tool Sequence Identity And Similarity (SIAS^10^). All sequences were edited with GeneDoc software 2.7.

### *In Silico* Analysis of Putative Promoter Sequences of Rice Tetraspanins

Nucleotide sequence 1 kb upstream of translational start site of rice tetraspanin genes were extracted from RGAP v7.0. The putative promoter sequences were analyzed for various *cis*-acting regulatory elements using New PLACE (A Database of Plant *Cis*-acting Regulatory DNA Elements^11^) and PlantCARE (Plant *Cis*-Acting Regulatory Element^12^) databases. Different classes of regulatory elements involved in tissue specificity and stress responsiveness were identified and their positions were marked.

### Plant Material, Growth Conditions and Stress Treatments

*Oryza sativa* L. sp. *indica* var. Pusa Basmati 1 (PB1) seeds were surface sterilized with 70% ethanol for 1 min, followed by 2% sodium hypochlorite for 20 min. After overnight soaking in water, seeds were grown hydroponically on rice growth media or RGM (Yoshida et al., 1976) for 7 days at 28 ± 2°C under 16 h light/8 h dark photoperiodic conditions. Seven-day-old rice seedlings were exposed to different abiotic stresses such as heat stress (42°C for ½, 1, 4, and 8 h), salinity stress (200 mM NaCl for 2, 6, 12, and 24 h), cold stress (4°C for 2, 6, 12, 24, and 48 h), water-deficit stress (imposed by 15% PEG-6000 for 2, 6, 12, and 24 h) and oxidative stress (10 mM H_2_O_2_ for 1, 4, 8, and 12 h). Similarly, several hormones such as ABA (100 μM), brassinosteroid (1 μM epibrassinolide), methyl jasmonate (100 μM), and gibberellic acid (100 μM) were also applied exogenously for 1, 3, 6, and 12 h. For simulating nutrient deprivation conditions 7-day-old rice seedlings grown in RGM [1.44 mM NH_4_NO_3_, 0.3 mM NaH_2_PO_4_, 0.5 mM K_2_SO_4_, 1.0 mM CaCl_2_, 1.6 mM MgSO_4_, 0.06 μM (NH_4_)_6_Mo_7_O_24_, 15 μM H_3_BO_3_, 8 μM MnCl_2_, 0.12 μM CuSO_4_, 0.12 μM ZnSO_4_, 29 μM FeCl_3_, 40.5 μM citric acid, pH 4.5-5.0] were deprived of nitrogen (-N) or phosphorous (-P) or potassium (-K) and sulfur (-S) for 12, 24, 48, and 72 h.

For tissue-specific expression profiling, different tissues such as shoots and roots of 7-day-old seedlings, leaves at young stage (15 Days After Transplanting or DAT), active tillering phase (40 DAT) and stem elongation phase (60 DAT), spikelets (80 DAT), young flag leaf (YFL; 70 DAT), mature flag leaf (MFL; 85 DAT). Fully expanded mature flag leaves were also collected at different stages of senescence, i.e., early stage or S1 (105 DAT), mid stage or S2 (120 DAT) and late stage or S3 (135 DAT) with 95–100%, 60–80%, and 40–60% total chlorophyll content, respectively. For all the treatments and stages appropriate controls were kept and the tissues were harvested, quickly frozen and stored at -80°C.

### RNA Isolation, cDNA Synthesis and Quantitative PCR

Total RNA was isolated following the modified protocol by Chomczynski and Sacchi (1987). The quantitative and qualitative analysis was carried out using spectrophotometer (Bio-Rad, USA) and 1.2% formaldehyde agarose gel, respectively. 2.5 μg of total RNA treated with DNaseI (New England Biolabs, USA) was reverse transcribed using iScript cDNA Synthesis Kit as per manufacturer’s instructions (Bio-Rad, USA). Quantitative PCR (qPCR) was performed with three biological replicates and two technical replicates using Sso fast Evagreen supermix (Bio-Rad, USA), appropriate primers and Master cycler RealPlex2 (Eppendorf, Germany). Melting curve analysis was performed to check the specificity of amplification with a particular set of primers. *eEF1α* (eukaryotic translation elongation factor 1 α; GenBank Accession #: AK061464) was employed as internal control for normalization. Relative fold change was calculated using ΔΔCT method as proposed by Livak and Schmittgen (2001). Hierarchical clustering analysis of relative fold change was performed to prepare a dendrogram and a heat map using Hierarchical Clustering Explorer v3.5 software. The nucleotide sequences of primers employed for gene expression analyses are provided in Supplementary Table S2.

For compiling expression profile of *OsTET* and *AtTET* genes using already available microarray datasets, heat maps were constructed. The datasets, employed for preparing rice *TET* genes expression profile, were retrieved from Genevestigator-https://genevestigator.com/gv/ (abiotic stress-specific profile) and RiceXpro-http://ricexpro.dna.affrc.go.jp/index.html (tissue-specific expression profile). Expression data on *Arabidopsis* plants treated with abiotic stresses was extracted from *Arabidopsis* eFP browser- http://bar.utoronto.ca/efp/cgi-bin/efpWeb.cgi.

### Subcellular Localization of Rice Tetraspanins

*Nicotiana benthamiana* (tobacco) transient assays were employed for determining the subcellular localization of OsTET proteins. For this cDNAs corresponding to *OsTET* genes were amplified using Phusion high-fidelity DNA polymerase (Thermo Scientific, USA) and cloned into pENTR-D/TOPO (Invitrogen, USA) followed by their mobilization into destination vector, pGWB541, to obtain OsTET-YFP fusion under the control of constitutive CaMV 35S promoter. All entry and expression clones were confirmed by DNA sequencing. For co-localization studies binary vector containing PM marker (PIP2A-CFP; ABRC catalog-pm-ck CD3-1001) was employed (Nelson et al., 2007). OsTET-YFP and PM marker constructs were mobilized into *Agrobacterium tumefaciens* strain GV3101. For transient assays, leaves from 4 to 6 week-old wild-type tobacco plants were infiltrated with the agrobacterial suspension harboring OsTET-YFP and PM marker as described by Voinnet et al. (2003). The infiltrated plants were marked and kept in a growth room at 25 ± 2°C. Fluorescence was visualized after 48–72 h of infiltration using TCS SP5 laser scanning confocal microscope (Leica, Germany). CFP and YFP signals were detected at 470–500 and 520–550 nm laser band width range with excitation at 433 and 514 nm lasers, respectively. All the images were further processed using Leica LAS AF Lite software. The nucleotide sequences of primers used for amplification of full length *OsTET* cDNAs are provided in Supplementary Table S3.

## Results

### Genome-wide Identification of Tetraspanin Gene Family Members in Rice Genome

To explore tetraspanin gene family in rice genome we performed a systematic analysis by employing two different approaches. First approach involved searching for the keyword ‘tetraspanin’ in two rice databases, Phytozome v10.3 and RGAP v7, which resulted in identification of 17 members belonging to tetraspanin family. The second approach was based on the prediction of TM helices in proteins using TMHMM v2.0 by which we were able to identify 11,404 membrane proteins in the RGAP rice database. Further, Perl scripts were designed to screen for the presence of four TM domains resulting in shortlisting of 569 proteins, which were further analyzed for the presence of canonical features of plant tetraspanins: two extracellular loops of unequal sizes, one intracellular loop, nine cysteine residues in EC2, presence of conserved ‘GCCK’ motif, short N- and C-terminal tails. The second approach identified 15 tetraspanin (*OsTET*) genes in rice. Comparison of the output from two approaches revealed that 15 members were commonly represented and were therefore considered as true tetraspanins, which was further confirmed by detailed analysis of protein sequence and domains. Two additional members identified by the first approach displayed presence of four TM, two ECL, one ICL, small N-, C- cytoplasmic tails but lacked highly conserved ‘GCCK’ motif in EC2. Moreover EC2 was found to be smaller in size than EC1 and it lacked conserved cysteine residues. For the abovementioned reasons, therefore these proteins were not considered as bonafide members of rice tetraspanin family. The 15 tetraspanins were subsequently named according to their locations on 12 rice chromosomes. The information on these 15 tetraspanin genes such as gene names, locus ID, number of introns, and details about the deduced protein are compiled and presented as **Table 1**.

**Table 1:** Details of rice tetraspanin genes.

Gene name	TIGR ID	RAP ID	Full-length cDNA accession ID	Full-length CDS (bp)	Number of introns	Accession number of rice T-DNA mutant	Predicted protein		
							Length (aa)	MW (kDa)	Putative localization
*OsTET1*	LOC_Os01g74570	Os01g0977100	-	866	1	-	321	35	PM
*OsTET2*	LOC_Os02g12750	Os02g0219800	AK242431	813	1	-	272	29.45	PM
*OsTET3*	LOC_Os02g49630	Os02g0728800	-	1143	4	-	381	41.43	PM
*OsTET4*	LOC_Os03g01012	Os03g0100020	AK073982	825	0	-	275	29.53	PM
*OsTET5*	LOC_Os03g63600	Os03g0853000	-	894	1	PFG_3A-15631.R	298	31.85	PM
*OsTET6*	LOC_Os03g63620	Os03g0853200	AK109128	858	1	PFG_4A-04052.L	286	30.86	Chloroplast
*OsTET7*	LOC_Os05g03140	Os05g0122800	AK065567	885	1	PFG_2D-11070.R	295	32.88	ER
*OsTET8*	LOC_Os05g03530	Os05g0126100	AK099070	867	1	PFG_1C-07632.R	289	31.14	PM
*OsTET9*	LOC_Os06g37510	Os06g0572400	AK070273	819	1	PFG_2A-30186.L	273	29.8	PM
*OsTET10*	LOC_Os06g44310	Os06g0653100	AK242956	810	1	PFG_2D-10796.L	270	29.49	PM
*OsTET11*	LOC_Os08g16050	Os08g0260600	AK108529	822	1	PFG_2B-60309.L	274	29.12	PM
*OsTET12*	LOC_Os08g34460	Os08g0443800	AK110630	834	0	PFG_K-00453.L	278	29.96	PM
*OsTET13*	LOC_Os09g25760	Os09g0425900	AK061801	831	1	FL054449	277	30.75	PM
*OsTET14*	LOC_Os10g35980	Os10g0503600	AK103857	813	10	PFG_2D-21433.R	271	30.62	PM
*OsTET15*	LOC_Os12g14580	Os12g0249100	-	933	1	-	311	34.13	PM

*Gene ID of each tetraspanin was retrieved from TIGR (The Institute for Genomic Research) and RGAP (Rice Genome Annotation Project) database. Length of coding sequence (CDS) of rice tetraspanin genes is presented along with the number of introns. Rice T-DNA mutants were identified from SIGNAL-RiceGE database v6.1. Molecular weights of tetraspanin proteins were predicted by using Geneious v6.0. Putative localizations of TET proteins were predicted with pSORT. Aa, amino acids; kDa, kiloDaltons; PM, plasma membrane; ER, endoplasmic reticulum; bp, base pairs; MW, molecular weight.*

### Chromosomal Distribution and Duplication Events Among Rice Tetraspanin Genes

With an aim to gain insight into the genomic organization of tetraspanin genes, their locations were mapped onto rice chromosome sequences. While 15 *OsTET* genes were unevenly distributed among 9 chromosomes, none of these genes could be mapped on chromosome 4, 7 and 11 (Supplementary Figure S1). Whereas chromosome 3 contained three *OsTET* genes, chromosomes 1, 9, 10, and 12 had only one *OsTET* gene each. To examine the inter-relationship of *OsTET* family genes we investigated for tandem and segmental gene duplications events. Only one pair of genes, *OsTET5* and *OsTET*6, separated by a genomic region of 7530 bp on chromosome 3, appeared to have arisen because of probable tandem duplication. *OsTET2* and *OsTET9* located on chromosome 2 and 6, respectively, were segmentally duplicated. *OsTET7* and *OsTET8* present on chromosome 5 were separated by a distance of 242 kb. *OsTET1* and *OsTET4* were located at the terminal portion of chromosome 1 and 3, respectively. Whereas, only six genes were present between *OsTET1* loci and the chromosome terminal, a single gene separated *OsTET4* from the end of chromosome 3 (Supplementary Figure S1).

### Gene Structure of Rice Tetraspanins

**FIGURE 1:**
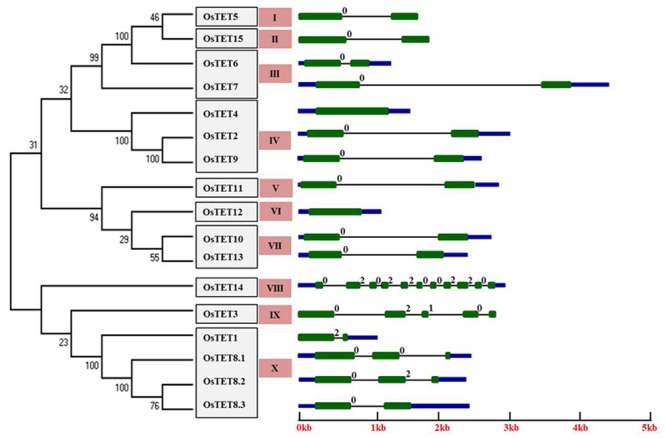
**Structure of rice tetraspanin genes and phylogenetic tree of predicted proteins.** An online tool, Gene Structure Display Server (GSDS; http://gsds.cbi.pku.edu.cn/), was used to draw tetraspanin gene structure. Green boxes indicate exons, black lines depict introns, upstream/downstream sequences are shown by blue boxes. Intron phases are indicated at exon-intron junctions. An unrooted neighbor-joining phylogenetic tree of rice tetraspanin proteins is shown on the left side. Numbers above branches indicate bootstrap percentage values. Clade numbers are indicated by salmon colored boxes. OsTET proteins were clustered based on significant bootstrap value (≥50%).

**FIGURE 2:**
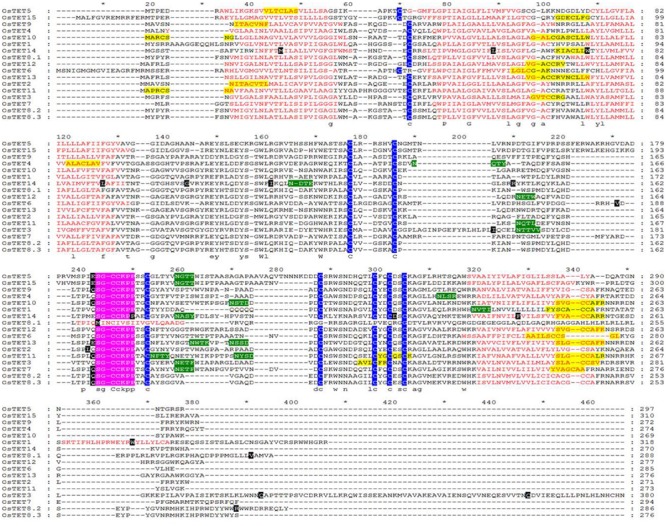
**Multiple sequence alignment of predicted rice tetraspanin proteins.** Protein sequences of rice tetraspanin proteins were retrieved from RGAP and were aligned using ClustalX 2.1. Red amino acids indicate transmembrane (TM) domains. Predicted palmitoylation and glycosylation sites are highlighted in yellow and green color, respectively. Highly conserved cysteine residues are shaded by blue color. Tetraspanin signature ‘SGCC(K/R)PP’ motif is shaded in pink color. Intron positions are shaded by black color. Sequences were edited with Genedoc software (http://www.nrbsc.org/gfx/genedoc/).

Understanding the gene structure of different members of a gene family provides information on the evolution of these genes. In fact tetraspanin superfamily in different organisms has been employed as an excellent system for studying intron evolution using phylogenomics approach (Garcia-Espana et al., 2009; Huang et al., 2010). Ten out of 15 rice tetraspanins exhibited conserved gene structure with two exons separated by a single intron and presence of short UTRs (**Figure 1**). *OsTET8* exhibited three splice forms, of which only *OsTET8.3* had conserved gene structure similar to that seen for most of the other tetraspanin genes, while the other two forms (*OsTET8.1* and *8.2*) contained two introns each. However, one of the spliced isoform, *OsTET8.1*, lacked ‘GCCK’ motif and other characteristic features of tetraspanin proteins (**Figures 1** and **2**). This analysis also suggests that rice tetraspanins have fewer introns relative to the average number of introns (3.9 introns/gene) reported in this species^13^. *OsTET14* contained a maximum of 10 introns followed by *OsTET3* that consisted of four introns. The sizes of introns in rice tetraspanin genes ranged from 95 to 4200 bp. *OsTET4* and *OsTET12* were predicted to be intron less genes (**Figure 1**).

The number and position of introns is generally conserved within members of a gene family (Fedorov et al., 1992). We found that in addition to the presence of a single intron in most of the rice tetraspanins position of the intron was also conserved. These introns were invariably present after the first second conserved cysteine residues in variable region of EC2. In a previous study, non-uniform distribution of intron phase has been reported in several plants, with phase 0 being highly represented and phase 2 being the least represented (Nguyen et al., 2006). After determining the gene architecture of 15 *OsTET* genes, we investigated distribution of the intron phase in these genes and found that majority of the *OsTET* genes contained phase 0 ancestral introns (introns that are present between two codons) followed by presence of phase 2 introns (introns present between the second and third bases of the codon). Among the 15 *OsTET* genes, only one (*OsTET3*) had a phase 1 intron (intron located between the first and second bases of the codon), along with two phase 0 and one phase 2 introns (**Figure 1**). All these observations support the earlier findings that phase 0 introns are most abundantly present in tetraspanin proteins (Garcia-Espana et al., 2009). Variation in the length of introns within family members is already reported and is believed to contribute to functional diversification of gene family members (He et al., 2012; Zhao et al., 2014). Considerable variation in the length of introns of 15 *OsTET* genes was found, with 95 bp in *OsTET14* as the shortest and 4200 bp in *OsTET7* as the longest intron, which suggests that members of *OsTET* family are functionally diverse.

### Phylogenetic Analysis and Protein Structure of Rice Tetraspanin Proteins

To determine the level of conservation and similarity among rice tetraspanin proteins, the predicted amino acid sequences of 15 OsTETs were aligned using ClustalX 2.1. On the basis of pairwise comparison of rice tetraspanins it was concluded that these proteins share an average of 34% identity and 48% similarity (Supplementary Figures S2–S4), which is comparable to that observed for *Arabidopsis* tetraspanin family of proteins (Boavida et al., 2013). An un-rooted phylogenetic tree was constructed using neighbor-joining method for members of rice and *Arabidopsis* tetraspanin protein family and the proteins were clustered together based on the significant bootstrap value of ≥50%. Among 15 OsTET proteins, only 10 proteins clustered with *Arabidopsis* proteins in orthologous clades. Our analyses revealed that OsTET5-OsTET7 and OsTET15 formed a cluster with AtTET3/AtTET4. While OsTET2/4/9 clade contained AtTET1/AtTET2 and OsTET1/OsTET8 clade consisted of AtTET5/AtTET6 (Supplementary Figure S5). Boavida et al. (2013) proposed that AtTET10 and AtTET13 are the founding members of *Arabidopsis* tetraspanin protein family. OsTET14 clustered closely with AtTET10, which signifies its ancient nature of origin, but a member corresponding to AtTET13 was not found in rice. AtTET7-AtTET9, AtTET11-AtTET17 formed separate clades, which indicated that these tetraspanins were specific to *Arabidopsis*. Similarly, OsTET3, OsTET10-OsTET13 did not cluster with *Arabidopsis* tetraspanins, which suggested that these tetraspanins were probably rice-specific (Supplementary Figure S5). We further constructed a phylogenetic tree for members of rice tetraspanin proteins only, which resulted in a total of 10 paralogous clades, of which clade IV consisted of a maximum of three OsTET proteins followed by clades III, VII, and X containing two members each (**Figure 1**). The tandemly duplicated tetraspanins, OsTET5 and OsTET6, were clustered in two different clades, I and III, respectively. The segmental duplicated members, OsTET2 and OsTET9, exhibited 100% bootstrap value indicating high level of conservation in their protein sequences (**Figure 1**).

The conserved domains of OsTET proteins were predicted so as to evaluate the extent of conservation among 15 members. Multiple sequence alignment of full length OsTET proteins revealed presence of four TM domains (predominantly 22 amino acids each), two extracellular loops of unequal sizes (EC1 and EC2) and highly conserved ‘GCC(K/R)’ motif, short N- and C- terminal cytoplasmic tails and small intracellular loop (ICL) as depicted in **Figure 2** and Supplementary Table S1. All OsTET proteins were found to contain one and nine conserved cysteine residues in EC1 and EC2, respectively. Animal and metazoan tetraspanins are known to possess conserved ‘CCG’ motif in EC2 domain (DeSalle et al., 2010). On the other hand plant tetraspanins are characterized by the presence of ‘GCCK/RP’ motif and few variants as seen in *Arabidopsis*. Interestingly we did not find any variant of ‘GCCK/RP’ motif in rice tetraspanin members. In fact we observed a relatively longer motif with sequence “SGCCK/RPP” in all the rice tetraspanin proteins. Most of the tetraspanins were predicted to contain potential post-translation modifications sites such *N*-glycosylation in EC2 region and palmitoylation of cysteine residues proximal to TMs, which are believed to play crucial role in protein–protein interactions. The TMs of all rice tetraspanins showed presence of conserved polar residues, which is another distinct feature of animal, metazoan and plant tetraspanins (**Figure 2**). The multiple sequence alignment and motif analyses revealed that rice tetraspanins possess characteristic features of plant tetraspanins and there exists high conservation in amino acid residues as well as motifs in these proteins, which is similar to that found in previously discovered plant tetraspanins.

### Expression Profiling of Rice Tetraspanin Genes in Different Tissues and during Flag Leaf Senescence

**FIGURE 3:**
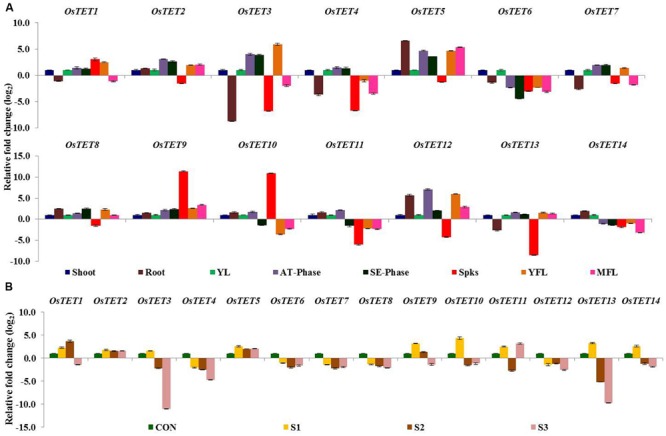
**Expression profiling of rice tetraspanin genes in different tissues of rice. (A)** Quantitative PCR analysis of transcript levels of rice tetraspanins in tissues namely shoot, root, young leaf (YL), active tillering phase (AT-Phase), stem elongation phase (SE-Phase), spikelets (Spks), young flag leaf (YFL), and mature flag leaf (MFL). For root tissue normalized fold change (log_2_ scale) was calculated relative to that in shoot tissue of 7-day-old seedlings. For tissues obtained from field grown plants fold change (log_2_ scale) was calculated relative to that in young leaf. **(B)** Quantitative PCR analysis of transcript levels of rice tetraspanins during progression of senescence in flag leaf of field-grown rice plants. CON: fully expanded MFL; 100% chlorophyll, S1: 80–90% chlorophyll; S2: 60–80% chlorophyll; S3: 40–60% chlorophyll. Normalized fold change (log_2_ scale) was calculated relative to that in MFL. For normalization *eEF-1α* was used as internal control. Three biological replicates and two technical replicates were included in the study. Error bars represent standard error (SE) of three independent biological replicates.

Two studies using *Arabidopsis* have implicated tetraspanin proteins in controlling developmental processes such as leaf venation, leaf pattering, root pattering, and floral organ development (Cnops et al., 2000, 2006; Olmos et al., 2003). On the basis of tissue- and domain-specific expression in *Arabidopsis*, Boavida et al. (2013) predicted involvement of tetraspanins in reproductive processes. With an aim to obtain the spatial-temporal expression profile of *OsTET* genes, we determined the expression levels of *OsTET* genes in various tissues: shoot and root tissues of 7-day-old seedlings, young leaf (YL), leaves at active tillering phase (AT-phase), leaves at stem elongation phase (SE-phase), spikelets (Spks), young (YFL) and mature fully-expanded flag leaf (MFL). Quantitative PCR was employed for determining the relative levels of *OsTET* transcripts in different tissues. However, only 14 out of the 15 predicted *OsTET* genes were amplifiable (Supplementary Figures S6 and S7) and therefore expression profiling studies were carried out for 14 out of 15 *OsTET* genes. The genes that exhibited ≥2-fold change in log_2_ scale in root tissue relative to the levels detected in shoot tissue of 7-day-old seedlings were considered as significantly regulated (**Figure 3A**). As compared to shoot tissue, three *OsTETs* (*OsTET5, 8, 12*) and four *OsTETs* (*OsTET3, 4, 7, 13*) were significantly up regulated and down regulated in root tissue, respectively. We also compared the relative levels of *OsTET* genes in field-grown young leaves (YL) with respect to the 7-day-old shoots. Notably, 10 out of 14 *OsTET* genes (*OsTET2-7, OsTET11-14*) were up regulated in YLs, of which *OsTET2* showed 21-fold change in its steady state levels (Supplementary Figure S8). For all other stages (AT-phase, SE-phase, Spks, YFL, and MFL) the transcript levels of *OsTET* genes were quantified relative to the levels present in YL. Expression of *OsTET6* declined in most of the tissues as compared to YLs. *OsTET2, 3, 5, 9, 11*, and *12* accumulated to higher levels in leaves at active tillering phase and their levels (except *OsTET11*) were sustained even during stem elongation phase (**Figure 3A**). Only three *OsTETs* (*OsTET1, 9*, and *10*) were highly expressed in the spikelets as compared to young leaf tissues. On the other hand six *OsTETs* (*OsTET3, 4, 6, 11, 12*, and *13*) exhibited significant decline in their transcript levels in spikelet tissue. Flag leaves harvested at both young and mature stages were used to investigate the expression pattern of *OsTETs*. It was observed that six *OsTETs* (*OsTET1, 3, 5, 8, 9*, and *12*) were highly expressive in young flag leaves, of which only *OsTET5, 9*, and *12* maintained their higher levels even in the MFL (**Figure 3A**).

Several tetraspanin genes in plants have been annotated as either senescence-associated genes (SAGs) or SAG-like, therefore it was reasonable to investigate the expression pattern of rice tetraspanins during flag leaf senescence. Three stages of senescence of fully expanded mature flag leaves of rice were included: early senescence (S1: total chlorophyll content was 80–90% of that in unsenesced flag leaf), mid senescence (S2: total chlorophyll content was 60–80%), and late senescence (S3: total chlorophyll content was 40–60%). Nearly 50% of rice tetraspanin genes, that included *OsTET1, 5, 9, 10, 11, 13*, and *14* exhibited high expression at S1 stage when compared to their expression in mature flag leaves (**Figure 3B**). With progression of senescence (stages S2 and S3), expression of several *OsTET* genes declined. However, *OsTET5* exhibited high expression levels (≥2-fold) at all the three stages of flag leaf senescence (**Figure 3B**). All these observations advocate involvement of tetraspanins in regulating development, including flag leaf senescence, in rice.

### Gene Expression Profiling of Tetraspanins under Different Abiotic Stresses, Nutrient Deprivation and Exogenous Treatment of Hormones

**FIGURE 4:**
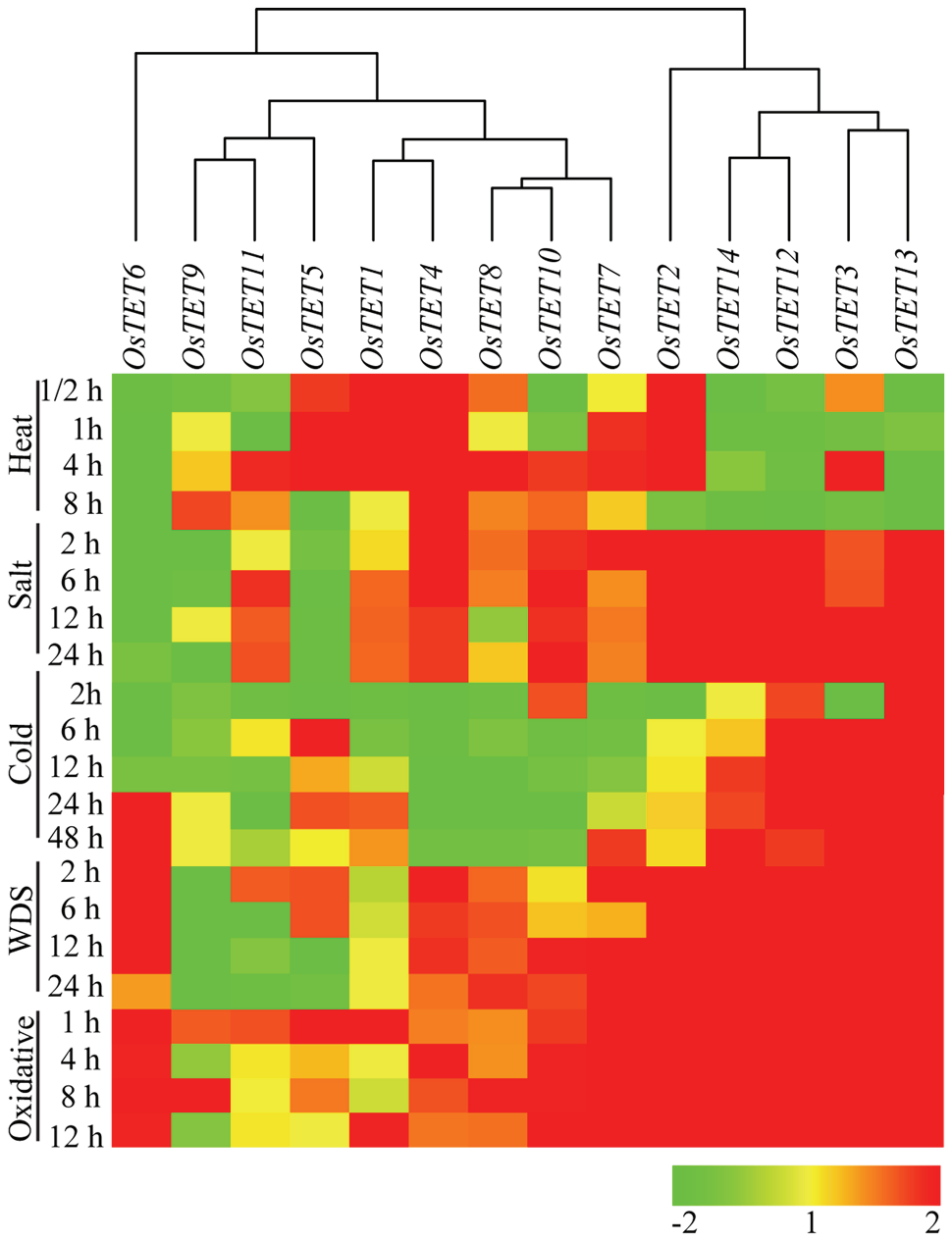
**Heat map representing expression profile of rice tetraspanin genes in rice seedlings exposed to different abiotic stresses.** Seven-day-old rice seedlings were exposed to different abiotic stresses such as heat stress at 42°C; salinity stress with 200 mM NaCl; water deficit stress (WDS) imposed by 15% PEG; cold stress at 4°C; oxidative stress with 10 mM H_2_O_2_ for different time durations as indicated on the left (duration in h). Expression levels of tetraspanin genes were determined by quantitative PCR and normalized fold change (log_2_ scale) was calculated relative to that in unstressed seedlings. For normalization *eEF-1α* was used as internal control. Three biological replicates and two technical replicates were included in the study. Hierarchical clustering analysis of relative fold change was performed to prepare a dendrogram and a heat map using Hierarchical Clustering Explorer v3.5 software.

To assess the role of tetraspanin genes in abiotic stresses, we initially performed a survey of publicly available gene expression atlas of rice seedlings exposed to different abiotic stresses^14^. Several of *OsTET* genes were found to be differentially expressed in seedlings challenged with abiotic stress conditions. However, as the expression data was available only for single duration of stress, therefore we designed experiments to determine the kinetics of *OsTET* gene expression. On exposure to supra-optimal temperature *OsTET1, 2, 4*, and *5* were substantially up regulated. The increase in their levels started as early as 30 min after heat stress and continued till 4 h of stress. Beyond this time-point, excluding *OsTET4*, expression of other genes either attained levels comparable to control conditions or declined (**Figure 4** and Supplementary Figure S9). On the other hand several *OsTET* genes (*OsTET6, 12, 13*, and *14*) were significantly down regulated during heat stress. On exposure of seedlings to high levels of salt, *OsTET2, 3, 4, 12, 13*, and *14* were highly induced. However, the expression levels of three *OsTETs* (*OsTET5, 6*, and *9*) declined considerably in high saline conditions.

On exposure of rice seedlings to low temperature conditions, *OsTET3, 12*, and *13* consistently exhibited high accumulation of their corresponding transcript, with the highest induction shown by *OsTET13* at 48 h (**Figure 4** and Supplementary Figure S9). Although levels of *OsTET6* transcript were low in cold stressed seedlings at earlier time points, there was significant up regulation at later time points of 24 and 48 h. Water-deficit conditions were imposed by transferring the rice seedlings to 15% PEG solution and the expression levels of *OsTET* genes were examined during stress. It was found that eight *OsTET* genes were upregulated as *OsTET2, 3, 4, 6, 7, 12, 13*, and *14* exhibited more than twofold change (on a log_2_ scale) in expression levels as compared to that in control conditions (**Figure 4** and Supplementary Figure S9). Oxidative stress imposed by H_2_O_2_ treatment resulted in up regulation of eight genes (*OsTET2, 3, 6, 7, 10, 12, 13*, and *14*), of which five (*OsTET2, 3, 12, 13*, and *14*) were also induced by salt treatment (**Figure 4** and Supplementary Figure S9). Noticeably these seven (*OsTET2, 3, 6, 7, 12, 13*, and *14*) out of eight genes exhibited consistent up regulation in their transcript accumulation at all time-points of oxidative stress tested in this study.

**FIGURE 5:**
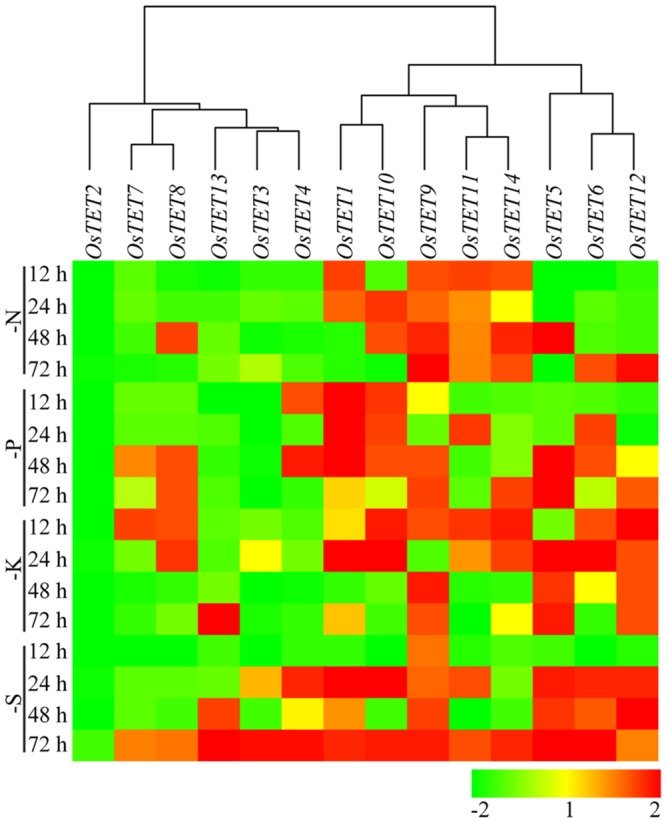
**Heat map representing expression profile of rice tetraspanin genes in rice seedlings exposed to nutrient deprivation.** Seven-day-old rice seedlings were grown in different nutrient deprivation media such as nitrogen deprivation (N), phosphorous deprivation (P), potassium deprivation (K), sulfur deprivation (S) for different time durations as indicated on the left (in h). Expression levels of tetraspanin genes were determined by quantitative PCR and normalized fold change (log_2_ scale) was calculated relative to that in unstressed seedlings. For normalization *eEF-1α* was used as internal control. Three biological replicates and two technical replicates were included in the study. Hierarchical clustering analysis of relative fold change was performed to prepare a dendrogram and a heat map using Hierarchical Clustering Explorer v3.5 software.

**FIGURE 6:**
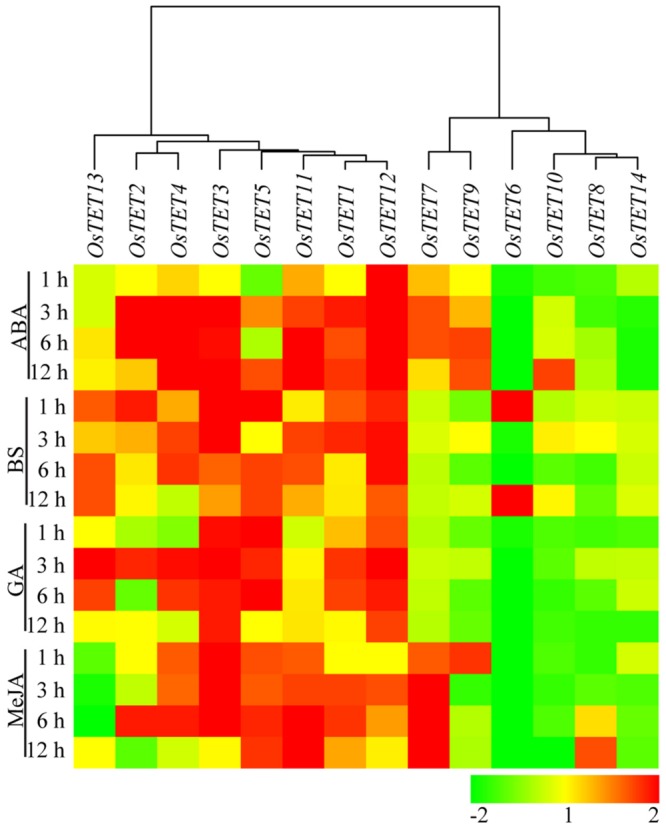
**Heat map representing expression profile of rice tetraspanin genes in rice seedlings exposed to different hormones.** Seven-day-old seedlings were exposed to exogenous hormones namely abscisic acid (ABA), brassinosteroids (BS), gibberellic acid (GA), and methyl jasmonate (MeJA) for different time durations (in h) as indicated on the left. Expression levels of tetraspanin genes were determined by quantitative PCR and normalized fold change (log_2_ scale) was calculated relative to that in untreated seedlings. For normalization *eEF-1α* was used as internal control. Three biological replicates and two technical replicates were included in the study. Hierarchical clustering analysis of relative fold change was performed to prepare a dendrogram and a heat map using Hierarchical Clustering Explorer v3.5 software.

Nutrient deprivation conditions were imposed by depleting growth medium with different essential elements that are normally required for optimal growth and development of rice (Qiu et al., 2009; Hur et al., 2012; Ma et al., 2012). Unlike the expression kinetics of *OsTET* genes under abiotic stress, very few genes were induced under nutrient deprivation conditions. While *OsTET1* was slightly induced in early time-points of nitrogen deprivation, *OsTET5* showed up regulation at later time-points only. *OsTET2* was significantly down regulated in all the nutrient deprivation conditions tested in this study. *OsTET1, OsTET5*, and *6* exhibited up regulation in sulfur-deprived seedlings (**Figure 5** and Supplementary Figure S10). Phytohormones are known to regulate developmental processes in plants and therefore it was worthwhile to examine whether exogenous application of these hormones affects the expression levels of *OsTET* genes in rice. Among 14 *OsTET* genes, seven *OsTET* genes (*OsTET2, 3, 4, 5, 7, 11*, and *12*) showed significant increase in their expression levels on exogenous application of plant hormones (**Figure 6** and Supplementary Figure S11). Out of 7 upregulated genes, *OsTET3* was noticeably induced by all the four hormone treatments. On the other hand *OsTET6* exhibited decline in expression when seedlings were treated with ABA, GA and MeJA (**Figure 6** and Supplementary Figure S11). Based on these observations it was concluded that while several *OsTET* genes were responsive to abiotic stress conditions, very few showed alteration in expression under nutrient deprivation or by hormone treatments.

**FIGURE 7:**
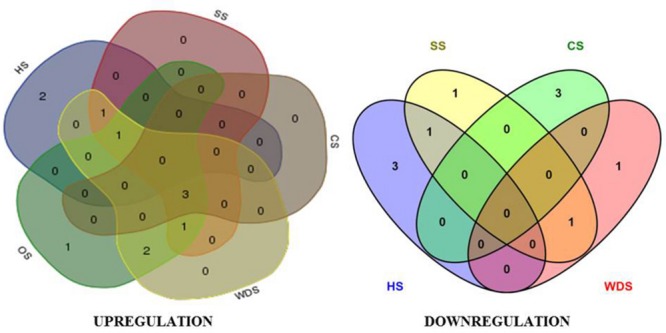
**Overlap in expression pattern of rice tetraspanin genes in various abiotic stresses.** Venn diagram representing overlap of *OsTET* genes exhibiting significant change (≥2-fold change in log_2_ scale) in expression among different abiotic stresses. Both upregulated and downregulated genes are shown. Five abiotic stresses: heat stress (HS), salinity stress (SS), cold stress (CS), water deficit stress (WDS), and oxidative stress (OS) were included in this study.

With an aim to characterize the overlaps in the expression pattern of members of *OsTET* gene family, we constructed Venn diagrams. Only the genes that exhibited consistent change in expression (twofold or more on log_2_ scale) at several time-points tested in our time kinetics study (**Figures 3** and **4**) were included in this analysis. We compared the differentially expressing *OsTET* genes in eleven different tissues (shoot, root, young leaf, active tillering phase, stem elongation phase, spikelets, YFL, MFL, S1, S2, and S3 stages of flag leaf senescence) versus those in five abiotic stresses (heat, cold, salinity, water-deficit, and oxidative stress) tested in this study. It was found that three (*OsTET8, 9*, and *11*) and two (*OsTET3* and *OsTET7*) *OsTET* genes were specifically induced and repressed in tissues, respectively (Supplementary Figure S12). A similar number of TET genes were either up regulated (*OsTET4, 6*, and 7) or down regulated (*OsTET5* and *OsTET9*) in all the abiotic stress conditions. Eight rice tetraspanin genes were found to be co-regulated in tissue as well as during abiotic stress (Supplementary Figure S12). Among five abiotic stress treatments only two (*OsTET1, 5*) and three *OsTET* (*OsTET12-14*) genes were specifically up regulated and down regulated during heat stress, respectively (Supplementary Figure S12). Interestingly the expression levels of three *OsTET* genes (*OsTET3, 12*, and *13*) were commonly induced by four abiotic stress treatments (SS, CS, WDS, and OS; **Figure 7**). During cold stress three *OsTET* genes (*OsTET4, 8*, and *10*) were specifically repressed followed by one each in salinity stress (*OsTET*5) and water-deficit stress (*OsTET11*). It is evidently clear that very few *OsTET* gene members are specifically regulated in either tissues or stress-treated seedlings. Nevertheless, several members of tetraspanin family in rice have evolved to function redundantly in development as well as during stress, pointing toward the biological significance of these proteins in plants.

### *In Silico* Analysis of Rice Tetraspanin Promoters for Putative *cis*-acting Elements

In the present study it is evidently clear that several of *OsTET* genes are spatio-temporally regulated and it is therefore pertinent to identify *cis*-acting regulatory elements within the promoter regions of these genes and correlate with their expression/function. The nucleotide sequence 1 kb upstream of translation initiation site was extracted and scanned for sequences of *cis*-elements using New PLACE and PlantCARE databases. The analysis resulted in identification of large number of putative *cis*-elements in the promoters of 15 *OsTET* genes. The data pertaining to six *cis*-acting elements, which are mainly associated with abiotic stress response or tissue specificity or hormone response (ABRE, HSE, MeJA response, LTR, POLLEN1LELAT52, and root motif-containing elements), is presented as Supplementary Figure S13. We found two temperature-responsive motifs in the promoters of *OsTET* genes: HSEs (heat shock elements) which are abundantly present in the promoters of majority of heat shock induced genes (Nover et al., 2001) and LTRs (low temperature response elements) which are associated with induction of cold-regulated genes (Dunn et al., 1998). The promoters of *OsTET1*, 2, *3, 9, 10*, and *12* were found to contain HSE, however, out of these six genes only *OsTET1* and two displayed induction by high temperatures in our expression studies (**Figure 4**). Similarly, though LTRs could be identified in the promoters of eight *OsTET* genes (*OsTET1, 2, 3, 7, 11, 12, 13*, and *14*), only three (*OsTET3, OsTET 12*, and *13*) were found to be responsive to cold stress (**Figure 4**).

ABRE or ABA-responsive elements are known to confer ABA-responsiveness to minimal plant promoters and we found that at least 4 *OsTET* genes (*OsTET4, 7, 9*, and *11*) contained ≥3 ABREs in their promoter regions. Furthermore we found that all these four genes exhibited significant transcript accumulation in response to exogenous application of ABA (**Figure 6**). It is known that multiple ABREs or ABRE in combination with coupling elements (CEs) or DREs (dehydration responsive elements) are involved in triggering induction by ABA (Narusaka et al., 2003; Zhang et al., 2005; Gomez-Porras et al., 2007). ABA is crucial in regulating several physiological, developmental and abiotic stress responses in plants (Yamaguchi-Shinozaki and Shinozaki, 1994; Fujita et al., 2011). Several of the ABA-responsive *OsTET* genes were found to be induced by various abiotic stresses such as salinity, cold, water-deficit, and oxidative stress in the present study (**Figure 4**). It would be interesting to look for other CEs and DREs in the promoter regions of *OsTET* genes, analyze the orientation and combination of these motifs and subsequently correlate with gene expression analyses. Although we found methyl jasmonate (MeJA)-responsive elements in upstream regions of several *OsTET* genes (*OsTET2, 4, 6, 7, 9, 10–12, and 14*) but among these only two genes (*OsTET7* and *11*) were significantly up regulated by application of methyl jasmonate (**Figure 6**).

Two tissue-specific motifs were also identified: root motif that confers specific expression in roots (Elmayan and Tepfer, 1995) and POLLEN1LELAT52 which is responsible for pollen-specific activation of genes (Filichkin et al., 2004). Root-specific elements were found in several *OsTET* genes (*OsTET1-5, 8–12, and 14*), with the maximum copies in *OsTET5* (10 root motifs) and this corroborates with high expression of *OsTET5* in root tissue. Although *OsTET12* promoter contained only one root motif the corresponding gene exhibited increased expression in root tissue when compared with shoot tissue. POLLEN1LELAT52 element was also identified in eleven OsTET gene promoters (*OsTET1-4, 6, 8–12*, and *15*), of which *OsTET1, OsTET9* and *10*, exhibited spikelet-specific expression (**Figure 3A** and Supplementary Figure S13). Boavida et al. (2013) have shown that tetraspanin genes are discretely expressed in male and female gametophytes of *Arabidopsis*. It would be worthwhile to study the expression levels of *OsTET* genes in male and female reproductive parts of rice. All these analyses confirm the presence of multiple *cis*-elements in the promoter regions of *OsTET* genes that could explain the complexity in their expression profile in different tissues, under various abiotic stress conditions and by hormone treatments.

### Subcellular Localization of OsTET Proteins in Transient Tobacco Assays

**FIGURE 8:**
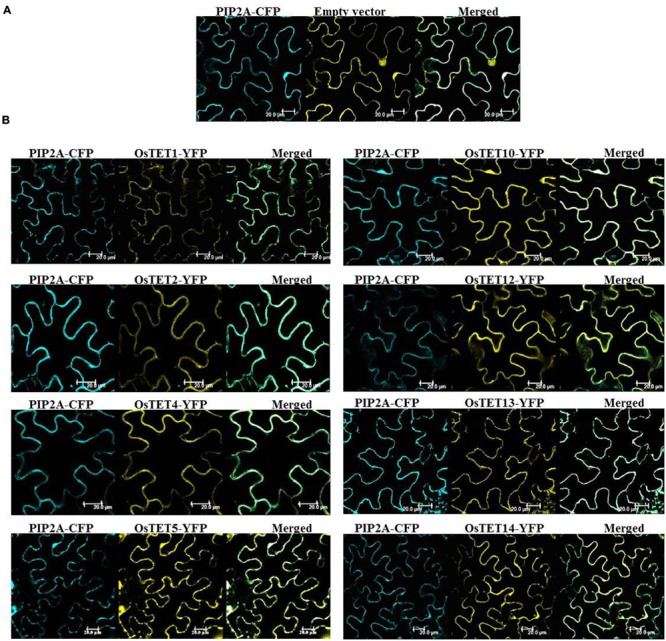
**Subcellular localization of rice tetraspanin proteins in tobacco epidermal cells.** The OsTETs fused to YFP were transiently expressed in tobacco (*Nicotiana benthamiana*) leaves. Localization in plasma membrane was confirmed by coexpression of PIP2A-CFP, a plasma membrane (PM) marker. **(A)** Colocalization of pGWB542 (empty vector) with PM marker. **(B)** Colocalization of OsTET-YFP proteins with PM marker. The merged fluorescence of marker and OsTET-YFP is shown at the right. Scale bar = 20 μm.

Subcellular localization studies are helpful in understanding the biological function as well as predicting molecular interactions of proteins in plants. *In silico* prediction of subcellular localization suggested that majority of OsTET proteins are targeted to plasma membrane, with two exceptions, of *OsTET6* and *OsTET7* genes that were predicted to reside in chloroplast, and endoplasmic reticulum, respectively. Transient expression of genes is a reliable, simple, and rapid approach for determining the subcellular localization of several plant proteins (Kokkirala et al., 2010). We employed model plant system *N. benthamiana* (tobacco) leaves for agroinfiltration of several OsTET-YFP fusion constructs. The PCR-amplified cDNAs corresponding to several *OsTET* genes were fused in-frame to YFP cDNAs under the control of constitutive cauliflower mosaic virus 35S (CaMV35S) promoter. These constructs were mobilized into *Agrobacterium* and were transiently expressed in tobacco leaves. Visualization of infiltrated tobacco leaves showed that all the OsTET proteins tested in this study distinctly accumulated at the periphery of tobacco epidermal cells which overlapped with the plasma-membrane marker protein, PIP2-CFP (**Figure 8B**). In the leaf tissues infiltrated with YFP alone (vector only), fluorescence was observed throughout the cell, i.e., in the nucleus as well as cytoplasm (**Figure 8A**). Based on the co-localization studies it is concluded that several OsTET proteins are specifically targeted to plasma membrane, which is also in agreement with prediction studies as well as previous studies on *Arabidopsis* TET proteins.

## Discussion

Tetraspanin genes encode for proteins that are components of large molecular complexes at the surface of the plasma membrane. These proteins together with their interacting partners form microdomains that modulate signaling cascades, thereby affecting diverse biological processes. With an exception of yeast, TET proteins are present ubiquitously in eukaryotes. With an aim to comprehend their biological roles in plants, we performed characterization of the rice tetraspanin family members.

### Rice Genome Encodes for 15 Tetraspanin Proteins

The availability of complete and annotated rice genome sequence was seemingly an appropriate starting point for identification of tetraspanin genes in rice. We devised a stringent strategy based on presence of all the canonical features in plant tetraspanin proteins and recognized 15 true tetraspanin members in rice genome. The *OsTET* loci were unevenly distributed throughout the rice genome as evident by their mapping onto only nine chromosomes. In contrast the 17 *Arabidopsis TET* (*AtTET*) loci reside on all five chromosomes, of which four members were present on chromosomes 1, 2, and 5 each (Boavida et al., 2013). With an exception of *OsTET5/6* that were clustered on chromosome 3, no other clusters of *OsTET* loci were found. Predictions revealed that *OsTET5/6* were present as tandemly duplicated loci, whereas *OsTET2/9* were segmentally duplicated. Gene duplications have acted as the fundamental force for the functional diversification and providing evolutionary novelty in plant genomes. Divergence occurs mainly due to mutations or conversions in coding sequence or regulatory region (Fawcett and Innan, 2011; Wittkopp and Kalay, 2012). We therefore aligned coding sequences and the respective upstream *cis*-regulatory regions of the two duplicated pairs (*OsTET2* vs. *OsTET9* and *OsTET5* vs. *OsTET6*). Interestingly no significant homology was found in the regulatory regions within members of the two pair (data not shown). However, we found 71 and 44% identity in the protein sequences of OsTET2/9 and OsTET5/6 pairs, respectively. Similarly, analysis of *AtTET* genes indicated that *AtTET11/16* residing on chromosome 1 were organized in tandem (analysis based on Du et al., 2013; Jiang et al., 2013). It would be interesting to correlate the level of sequence conservation with the spatio-temporal expression within members of duplicated pairs.

Gene architecture studies showed that majority of the *OsTET* genes contained a single ‘phase 0’ intron, with a conserved intron/exon junction. Abundance of phase 0 introns in rice tetraspanin gene family is in agreement with earlier findings showing phase 0 introns as the most abundant and phase 2 as the least abundant in tetraspanin genes of several organisms (Fedorov et al., 1992). Due to presence of 10 introns (a feature considered to be present in ancient genes) and its homology with *AtTET10* (considered as one of the founding members of *TET* gene family in *Arabidopsis*), *OsTET14* appears to be the founding member of rice tetraspanins. Detailed phylogenetic analysis of tetraspanin evolutionary origin and organization performed with genomes of metazoans, fungal, animal, and plants suggested that tetraspanins are derived from single or few ancestral gene(s) through sequence divergence (Garcia-Espana et al., 2008). Biasness in position of intron with respect to the protein sequence has been reported (Fedorov et al., 2002) in tetraspanin genes and our analysis also revealed that majority of rice tetraspanin family members had insertion of intron in the region coding for EC2 domain. Our results are in concordance with Garcia-Espana et al. (2009) who studied intron evolution in tetraspanins and found that EC1 and EC2 domains are the hotspots for insertion of new introns.

### Rice Tetraspanin Proteins Contain Conserved Domains

The availability of complete protein sequences of *Arabidopsis* and rice tetraspanins enabled us to perform a detailed phylogenetic analysis of OsTET with AtTET proteins. Notably, AtTET10, one of the *Arabidopsis* founding tetraspanin protein clustered with OsTET14. Several AtTET members were clustered as an outgroup because they did not group with any OsTET protein, which indicated that they could be specific to *Arabidopsis*. Similarly, OsTET3, OsTET10-13 formed separate clades from AtTET members suggesting that these members are specifically present in rice or possibly only in monocots. To confirm this, we further analyzed the phylogenetic relationship of rice tetraspanins with other plant tetraspanins presented by Boavida et al. (2013) and found that most of the *Sorghum bicolor* TET proteins clustered with OsTET proteins. Two SbiTETs (Sb04g029140, Sb10g009940) clustered with OsTET3, which hinted that OsTET3 is a monocot-specific tetraspanin. However, OsTET10-13 clustered with TET proteins from other dicots such as *Glycine max, A. lyrata, Vitis vinifera*, and *Populus trichocarpa* and are therefore not rice specific. Phylogenetic analysis of only OsTETs members resulted in 10 different clusters, wherein two segmental duplicates clustered closely in clade IV with 100% bootstrap value. The two tandem duplicated members, OsTET5 and 6, were separately placed in clades I and III, respectively. OsTET3, 5, 11, 12, 14, and 15 were represented as singletons in their respective clades. Rice tetraspanins share an average of 34% identity and 48% similarity in their amino acid sequences. The segmentally duplicated pair of OsTET2 and OSTET9, showed highest level of identity (83.4%) and similarity (90.5%) and were therefore adjacently placed in clade IV. On the other hand, OsTET3 and 6 pair exhibited 22.2% identity and 30.71% similarity, and were placed in clade IX and III, respectively.

A highly conserved ‘CCG’ motif within the EC2 domain is the hallmark of TET family members in animals and metazoans (Stipp et al., 2003). Plant TETs differ from animal and metazoan TETs as they contain signature ‘GCCK/RP’ motif (Wang et al., 2012). In fact ‘GCCK/RP’ motif is present in tetraspanins of an ancient vascular plant *Selaginella moellendorffii*, which suggests early appearance of this motif in plant ancestry followed by its reorganization (Garcia-Espana and Desalle, 2009). Variants of ‘GCCK/RP’ motif, such as ‘YCCAO’ or ‘GCCM/NR/P,’ are present in two members each of *Arabidopsis* TET family (Boavida et al., 2013). Noticeably a slightly longer conserved motif ‘SGCCK/RPP’ was present in EC2 domain of rice. Closer examination of 17 AtTET proteins revealed that at least eight of these contained conserved motif identical to that found in rice. The cysteine residues present in conserved motif mediate correct folding and stabilization of EC2. It has also been proposed that the conserved region of EC2 mediates homodimerization of TETs in membrane complexes, whereas variable region is responsible for specific binding with interacting protein partners (Stipp et al., 2003).

Amongst different domains of OsTET proteins, the two TM domains, TM2, and TM3, showed highest identity with an average 43.5 and 42.6%, respectively. The high level of identity between TM domains observed in tetraspanin proteins of rice and other organisms is crucial for the hydrophobic interactions, which are required for assembly of the tetraspanin web. While EC2 domain within OsTET proteins was more conserved (average 37.6% identity) as compared to EC1 (average 22.4% identity), the amino acid sequence of their short cytoplasmic C-terminal tail (average 14.2% identity) was highly variable. It is likely that the variability in C-terminus tail provide additional functional diversification by either directing subcellular localization or by its interaction with different partner proteins. OsTETs also consisted of a single conserved cysteine residue in EC1 as found in other plant tetraspanin members. Little is known about the molecular function of EC1 except a study by Masciopinto et al. (2001), which demonstrated that EC1 is required for optimal expression of EC2 on cell surface in a human tetraspanin protein, CD81. It will be worthwhile to investigate whether a similar role of EC1 exists in plants and whether the conserved cysteine residue is contributing to its function. Several potential palmitoylation and glycosylation sites predicted in majority of OsTET proteins, are likely to specify primary and secondary interactions within tetraspanin web. Based on these findings it is evident that OsTET proteins contain conserved domains and their further analysis will be helpful in linking the structural features of OsTET proteins with specific functions.

### Spatio-temporal Changes in Expression Suggest Functional Diversification of Rice Tetraspanin Family Members

Survey of the already available microarray-based expression data in *Arabidopsis* showed that few *AtTET* genes are differentially regulated by abiotic stress treatments in shoot and root tissues (Supplementary Figure S14). Similar analyses of rice microarray datasets indicated tissue- and abiotic stress-specific expression pattern of few *OsTET* genes (Supplementary Figure S15). With an aim to predict the biological function of OsTETs, detailed spatio-temporal expression analysis was carried out. Transcripts of three genes accumulated to higher levels in root tissue whereas transcripts of four genes were specifically present in active tillering phase. At the same time steady state levels of three genes were high in the spikelets. Flag leaf in cereals is the one of the most important leaves, which contributes to at least 50% of photosynthesis required for grain filling and yield (Sylvester-Bradley et al., 1990). Therefore studying the molecular changes that take place during its development and senescence would provide useful insights into mechanisms that control grain production. Six *OsTETs* were detected at higher levels in YFL, of which only four maintained their levels in MFL. The onset of senescence in flag leaves resulted in upregulation of seven *OsTETs*, of which *OsTET5* was conspicuously detected even at later stages of senescence. Except spikelets *OsTET5* and *OsTET12* were expressed at higher levels in all tissues indicating that might play a more generalized role in plant development. Members of segmentally duplicated gene pair, *OsTET2* and *9* co-expressed in several tissues whereas tandemly duplicated genes, *OsTET5* and *6*, exhibited inverse expression profile in most of the tissues. In addition to the sequence alignment that showed OsTET5 and 6 to be structurally diverse, their distribution in tissues indicates that they are functionally diverse as well. Overall *OsTETs* are widely expressed in different tissues of rice at different developmental stages, which may reflect their fundamental requirement in defining plant growth and development.

Perception of signals at the cell surface is one of the important steps in defining abiotic stress responses in plants. We studied the expression pattern of *OsTET* genes during various abiotic stress, nutrient deprivation, and hormone treatments. Among the five abiotic stresses studied *OsTET2* was induced by all stresses, except low temperature conditions. Similarly, *OsTET12* and *13* were upregulated by all, except high temperature stress. While *OsTET1* and *5* were specific to heat stress, *OsTET10* was slightly induced by oxidative stress only. Expression of *OsTET5, 8*, and *12* was specific to roots and it was conceivable that these genes are responsive to salinity or water deficit stress. *OsTET12*, but not *OsTET5* and *8*, was responsive to both the stresses in whole seedlings. As we pooled root and shoot tissue, wherein shoot tissue was predominantly represented, it is likely that stress mediated induction of *OsTET5* and *8* would be observed if their expression is analyzed in root tissue only. It will be interesting to study the expression profile of these *OsTET* genes separately in root and shoot tissue after stress imposition. In contrast to the coexpression of *OsTET2* and *9*, observed in a majority of tissues the transcripts of these genes were inversely regulated in almost all the examined abiotic stresses. On the other hand tandemly duplicated genes *OsTET5* and *6*, were seemingly coexpressed in salinity, oxidative stress, and sulfur deprivation. Future investigations on the factors regulating these genes in different tissues and stress conditions will be beneficial in delineating the mechanisms responsible for their conditional coexpression. Based on the expression studies it can be concluded that *TET* genes in rice underwent functional diversification with respect to development and abiotic stress conditions.

### Several Rice Tetraspanin Proteins Preferentially Associate with Plasma Membrane

To gain insights into the function of rice tetraspanins we performed subcellular localization of several OsTETs using tobacco transient assays. All the eight OsTETs (OsTET1, 2, 4, 5, 10, 12, 13, and 14) tested in this study were localized to the plasma membrane. This is in agreement with *Arabidopsis* TETs and mammalian TETs, which are preferentially associated with plasma membrane. However, few *Arabidopsis* TETs are known to accumulate in cytoplasmic organelles such as endoplasmic reticulum or ER (Boavida et al., 2013). The subcellular targeting prediction of OsTET7 suggested its localization to ER, however, it needs to be validated experimentally. It is possible that OsTET7 is localized in membranes of the ER and play a role in protein trafficking. Localization at cell surface is necessary for organizing tetraspanin microdomains and mediating cell-to-cell interactions. Boavida et al. (2013) demonstrated cell-specific accumulation of TETs in reproductive tissues of *Arabidopsis*, which provided evidence for their crucial role in reproductive development. Similar studies for rice tetraspanins would provide valuable information on their probable biological function.

TET proteins are dynamic in nature with respect to their molecular interactions and biological functions. The importance of TET proteins is highlighted by the functional redundancy among these proteins. Yeast split ubiquitin interaction assays confirmed AtTET proteins interact physically leading to formation of homomers or heteromers (Boavida et al., 2013). The functional redundancy could thus be attributed to multiplicity in TET–TET interactions, wherein association with specific interacting partners in microdomain imparts discrete biological functions to these proteins. Human CD151 is tightly associated with the integrins to mediate integrin-dependent cell adhesion activities (Levy et al., 1998; Berditchevski, 2001; Boucheix and Rubinstein, 2001). Potential association of tetraspanin with other partners such as immunoglobulin superfamily proteins, proteases, signaling enzymes, GPCRs, cadherins, and proteoglycans has been hypothesized (Hemler, 2005). Proteomic approaches for purification of tetraspanin microdomains complexes under different conditions or in different tissues followed by the identification of the components would contribute to the elucidation of molecular and functions of these proteins.

Collectively, the results obtained in the present study provide valuable insights into the genomic organization, phylogenetic relationship, protein structure, and functional significance of rice tetraspanin proteins. Comprehensive expression profiling of OsTETs suggests that they are crucial in regulating plant development and defining plant’s response to environmental challenges. Based on our findings it is can be envisaged that these genes are potential candidates for manipulating stress tolerance in plants.

## Author Contributions

SK-A conceptualized, designed and supervised the project. BM carried out all the experiments and *in silico* analyses. BM and SK-A wrote the manuscript. MA regularly discussed the experiments, analyzed the results, provided useful suggestions during the project and critically revised the manuscript. All authors read and approved the final manuscript.

## Conflict of Interest Statement

The authors declare that the research was conducted in the absence of any commercial or financial relationships that could be construed as a potential conflict of interest.

## Footnotes

1 http://rice.plantbiology.msu.edu/

2 http://www.phytozome.net/

3 www.cbs.dtu.dk/services/TMHMM/

4 http://viewer.shigen.info/oryzavw/maptool/MapTool.do

5 http://chibba.agtec.uga.edu/duplication/index/locus

6 http://rice.plantbiology.msu.edu/

7 http://www.arabidopsis.org

8 http://smart.embl-heidelberg.de/

9 www.cbs.dtu.dk/services/NetNGlyc/

10 http://imed.med.ucm.es/Tools/sias.html

11 www.dna.affrc.go.jp/PLACE/

12 http://bioinformatics.psb.ugent.be/webtools/plantcare/html/

13 http://rice.plantbiology.msu.edu/analysesfacts.shtml

14 https://genevestigator.com/gv/
