# Supplementary material for: Comprehensive Expression Profiling of Rice Tetraspanin Genes Reveals Diverse Roles During Development and Abiotic Stress
Source: Front Plant Sci. 2015 Dec 11;6:1088. doi: 10.3389/fpls.2015.01088 (PMC4675852; doi:10.3389/fpls.2015.01088)
Supplement: Supplementary file 1 [file Table_1.DOCX]

**Supplementary Table 1. Details of domains identified in rice tetraspanin proteins.** The predicted length of domains identified in OsTET proteins is shown. Transmembrane domains of OsTET proteins were predicted with SMART tool (http://smart.embl-heidelberg.de/). Potential palmitoylation sites were predicted with CSS-Palm v2 and NetNGlyc 1.0 Server (http://www.cbs.dtu.dk/services/NetNGlyc) was employed to identify potential N-glycosylation sites. N-tail: N-terminus cytoplasmic tail, TM: transmembrane domain, EC: extracellular loop, IL: intracellular loop, C-tail: C-terminus cytoplasmic tail, aa: amino acids.

| **Protein** | **N-Tail**  **(aa)** | **TM1**  **(aa)** | | **EC1**  **(aa)** | | **TM2**  **(aa)** | | **IL**  **(aa)** | | **TM3**  **(aa)** | | **EC2**  **(aa)** | | **TM4**  **(aa)** | | **C-Tail**  **(aa)** | | **No. of glycosylation sites** | | **No. of palmitoylation sites** | |
| --- | --- | --- | --- | --- | --- | --- | --- | --- | --- | --- | --- | --- | --- | --- | --- | --- | --- | --- | --- | --- | --- |
| OsTET1 | 18 | 22 | | 14 | | 22 | | 6 | | 22 | | 127 | | 22 | | 32 | | 1 | | 2 | |
| OsTET2 | 6 | 22 | | 14 | | 22 | | 5 | | 22 | | 142 | | 22 | | 14 | | 1 | | 1 | |
| OsTET3 | 6 | 22 | | 16 | | 22 | | 6 | | 22 | | 157 | | 22 | | 107 | | 2 | | 4 | |
| OsTET4 | 4 | 22 | | 14 | | 22 | | 6 | | 22 | | 141 | | 22 | | 18 | | 2 | | 2 | |
| OsTET5 | 6 | 22 | | 11 | | 22 | | 12 | | 22 | | 163 | | 22 | | 16 | | 1 | | 2 | |
| OsTET6 | 25 | 22 | | 9 | | 22 | | 12 | | 22 | | 139 | | 22 | | 9 | | 2 | | 2 | |
| OsTET7 | 6 | 22 | | 14 | | 22 | | 8 | | 22 | | 147 | | 22 | | 28 | | 2 | | 1 | |
| OsTET8.1 | 8 | 22 | | 13 | | 22 | | 6 | | 22 | | 66 | | 22 | | 103 | | - | | - | |
| OsTET8.2 | 8 | 22 | | 15 | | 22 | | 6 | | 22 | | 127 | | 22 | | 47 | | - | | 1 | |
| OsTET8.3 | 8 | 22 | | 15 | | 22 | | 6 | | 22 | | 127 | | 22 | | 32 | | - | | 1 | |
| OsTET9 | 6 | 22 | | 14 | | 22 | | 6 | | 22 | | 138 | | 22 | | 17 | | 1 | | 1 | |
| OsTET10 | 8 | 22 | | 14 | | 22 | | 6 | | 22 | | 136 | | 22 | | 14 | | 1 | | 3 | |
| OsTET11 | 6 | 22 | | 19 | | 22 | | 6 | | 22 | | 137 | | 22 | | 13 | | 4 | | 3 | |
| OsTET12 | 6 | 22 | | 14 | | 22 | | 6 | | 22 | | 138 | | 22 | | 22 | | 1 | | 2 | |
| OsTET13 | 6 | | 22 | | 14 | | 22 | | 6 | | 22 | | 137 | | 22 | | 22 | | 2 | | 2 |
| OsTET14 | 11 | | 20 | | 9 | | 22 | | 11 | | 22 | | 133 | | 22 | | 19 | | 2 | | 2 |
| OsTET15 | 20 | | 22 | | 14 | | 22 | | 12 | | 22 | | 133 | | 22 | | 20 | | 1 | | 1 |
