# Supplementary material for: Comprehensive Expression Profiling of Rice Tetraspanin Genes Reveals Diverse Roles During Development and Abiotic Stress
Source: Front Plant Sci. 2015 Dec 11;6:1088. doi: 10.3389/fpls.2015.01088 (PMC4675852; doi:10.3389/fpls.2015.01088)
Supplement: Supplementary file 2 [file Table_2.DOCX]

**Supplementary Table 2. Nucleotide sequences of primers used for quantitative PCR.**

| **Gene name** | **Forward primer or FP (5’----3’)** | **Reverse primer or RP (5’----3’)** |
| --- | --- | --- |
| *OsTET1* | CTACGGGTGCGACTCGTGCAA | GAGGAGGACGACGTTGAGGATG |
| *OsTET2* | GCCCTCTGCTTCAATTCAGCTTCA | TGGCCATGAATCTTAACTACTACTCC |
| *OsTET3* | CTCTGTGACGTGACAACCGTGC | AGTTTCGCCTGACGGTGTCCA |
| *OsTET4* | AGCTCTGCTACTCCTGCTCCT | TACGTGTAGCCCTGCCTGTAG |
| *OsTET5* | AATGCCCGAGAGTACAGCCTCAG | GTGTCTCTTGGCCCACCTTTCG |
| *OsTET6* | GCTGCAAGCCACCATCATCGT | GAGGAAGCCTGCCTTGCATGA |
| *OsTET7* | GCAGACGCTTTGCTTCCAGTGT | CCTTGCCATACCAAACGGCTCG |
| *OsTET8* | TGATGGAGAAGGTGAGGGAGGA | CCACCATCGCCACCAGTAGTAG |
| *OsTET9* | CGGGGTGAGGTGTGATTGGTAGA | GACTACCGCCTCGACGGCTAC |
| *OsTET10* | GGCGATGTATAGGTTCTTAGGTCG | AGCAACCATGCTTCAACTAGCAGC |
| *OsTET11* | GCCTCCTACACCCAGCAACTAC | GGCGTTGACGAAGGCGATCTT |
| *OsTET12* | CGGTTTCAAAAGTTTGAGTACGAGC | GCTAAACACGGTGTCAGAAGACTAG |
| *OsTET13* | TGCTACAACTGCCAGTCGTGCAA | GGTTGTTCCTGAACGCGCAGCA |
| *OsTET14* | CCAGTCAGCACGAATGTCGACT | AGCACGCGACGAAGTAGACG |
| *eEF1α* | GGCCATGGCACTCGTTGCATT | TACCCGCATTCCACAACAGCC |
