# Supplementary material for: Comprehensive Expression Profiling of Rice Tetraspanin Genes Reveals Diverse Roles During Development and Abiotic Stress
Source: Front Plant Sci. 2015 Dec 11;6:1088. doi: 10.3389/fpls.2015.01088 (PMC4675852; doi:10.3389/fpls.2015.01088)
Supplement: Supplementary file 3 [file Table_3.DOCX]

**Supplementary Table 3. Nucleotide sequences of primers used for amplification of full length *OsTET* cDNAs.**

| **Gene name** | **Forward primer or FP (5’----3’)** | **Reverse primer or RP (5’----3’)** |
| --- | --- | --- |
| *OsTET1* | CACCATGCTGCTCCTCGTGCTGGCCCTGC | CAAGCTAGCTAGAGAAGTAGAGATGGATGATTGCTC |
| *OsTET2* | CACCATGGCGGTGAGCAACAACATCACGGCGTGCGTGACGCTGA | CCACTTGTAGCGGCGGAAGAGGGCCTCGGCCT |
| *OsTET4* | CACCATGGCCCTCAACTATGTGGGC | CGTGTAGCCCTGCCTGTAGCGG |
| *OsTET5* | CACCATGACGCCAGAAGACCGAGC | TCGTGATCTTCCAGTATTATTATTCCCAGTTGCTTG |
| *OsTET10* | CACCATGGCGCGGTGCAGCAACGGC | TTTCCAAGCCGGGTACGAGTTGTCC |
| *OsTET12* | CACCATGGCGTTCCGGCTCAGCAACAACGTG | GGCGTATCCGGCCTGCTTCCACCCG |
| *OsTET13* | CACCATGGCGTTCCGGCTGAGCAACAGCCTG | GGCGTATCCGCCCTTCCACGCGGCAC |
| *OsTET14* | CACCATGGGTAGTAGCACAAGCGCCTTC | TGCATGCCATCTTGTAGGAACCTTGG |
