# Supplementary material for: Comprehensive Expression Profiling of Rice Tetraspanin Genes Reveals Diverse Roles During Development and Abiotic Stress
Source: Front Plant Sci. 2015 Dec 11;6:1088. doi: 10.3389/fpls.2015.01088 (PMC4675852; doi:10.3389/fpls.2015.01088)
Supplement: Supplementary file 4 [file Presentation_1.PDF]

Supplementary Figures

Comprehensive expression profiling of rice tetraspanin genes reveals diverse roles during development and abiotic stress

Balaji M[1], Manu Agarwal[2], Surekha Katiyar-Agarwal[1*]

Author Affiliations:

[1] Department of Plant Molecular Biology, University of Delhi South Campus, New Delhi 110021, India.

[2] Department of Botany, University of Delhi, Delhi 110007, India.

***Correspondence:** Dr. Surekha Katiyar-Agarwal, Lab No. 314, Department of Plant Molecular Biology, University of Delhi South Campus, Benito Juarez Road, New Delhi-110021, INDIA.

katiyars@south.du.ac.in, katiyarsurekha@gmail.com, Tel.: +91-11-24113915

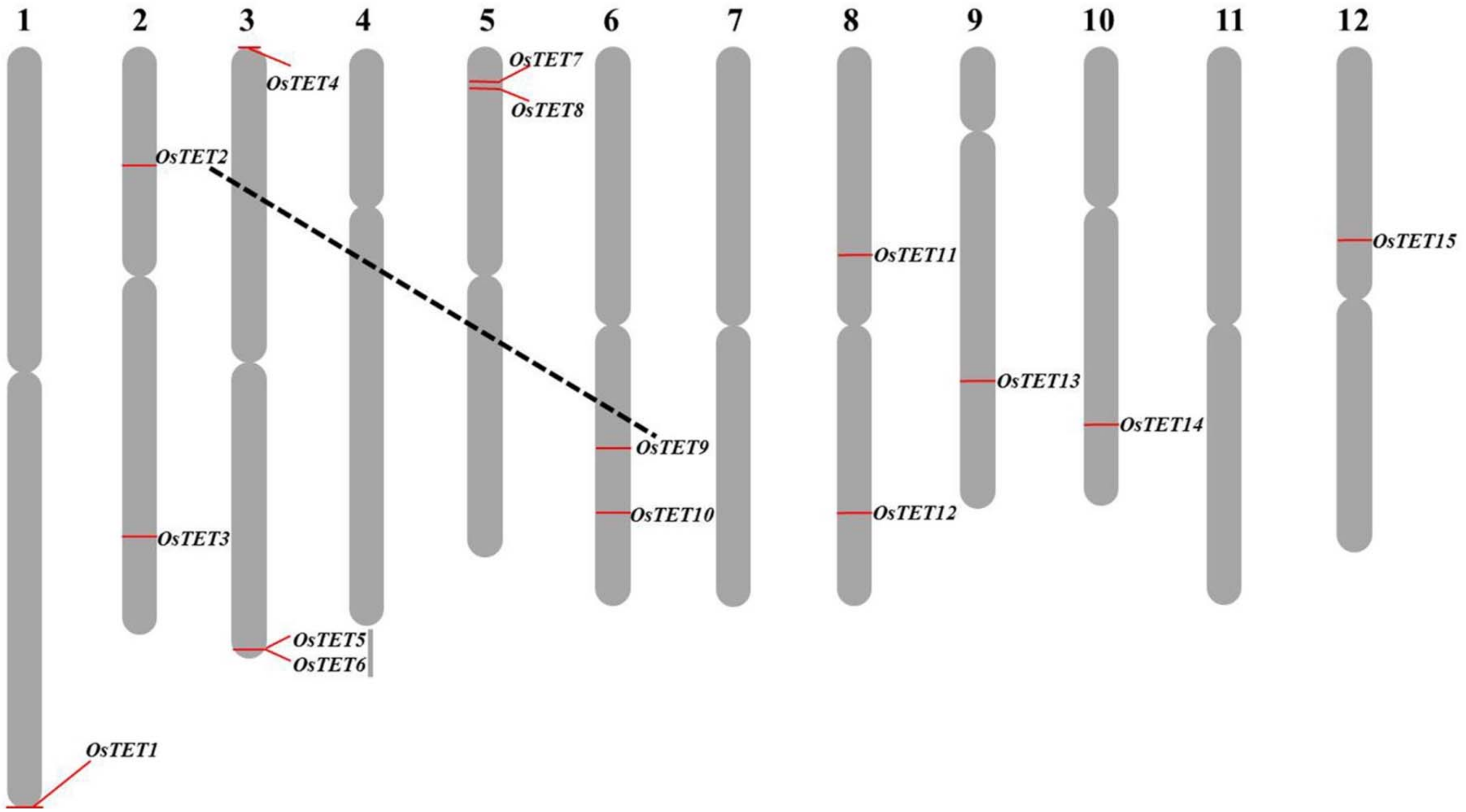

Supplementary Figure 1. Chromosomal distribution of rice tetraspanin genes. Mapping of 15 rice tetraspanin genes was performed onto 12 chromosomes of rice using chromosome map tool (http://viewer.shigen.info/oryzavw/maptool/MapTool.do) and the diagram was drawn on the basis of output generated. The chromosome numbers are shown on the top. Vertical grey and black dotted lines depict predicted tandem and segmental duplication of tetraspanin genes, respectively.

Supplementary Figure 2 (A)

OsTET indica and japonica proteins identity matrix

**OsITET1**	100													
**OsITET2**	32.22	100												
**OsITET3**	35.58	30	100											
**OsITET4**	36.86	47.03	31.02	100										
**OsITET5**	21.88	25.18	23.48	24.81	100									
**OsITET6**	22.93	25.18	24.37	23.72	54.48	100								
**OsITET7**	36.39	34.07	37.01	37.95	33.67	39.78	100							
**OsJTET1**	96.97	32.22	35.58	36.86	21.88	22.93	36.39	100						
**OsJTET2**	31.98	100	30.14	46.69	25	25	34.19	31.98	100					
**OsJTET3**	34.56	30.37	98.57	31.75	22.22	24.37	35.37	32.39	30.51	100				
**OsJTET4**	36.72	47.03	30.90	100	24.72	23.63	37.81	36.72	46.69	31.63	100			
**OsJTET5**	22.48	25.55	24.19	25.54	98.31	54.12	34.01	22.48	25.36	22.81	25.45	100		
**OsJTET6**	23.42	26.29	24.55	24.45	54.54	99.64	39.51	23.42	26.10	24.12	24.36	54.19	100	
**OsJTET7**	36.27	34.07	37.01	37.95	33.55	39.78	100	36.27	34.19	35.25	37.81	33.89	39.51	100
	**OsITET1**	**OsITET2**	**OsITET3**	**OsITET4**	**OsITET5**	**OsITET6**	**OsITET7**	**OsJTET1**	**OsJTET2**	**OsJTET3**	**OsJTET4**	**OsJTET5**	**OsJTET6**	**OsJTET7**

Supplementary Figure 2 (A). Rice indica and japonica tetraspanin proteins identity matrix. OsTET1-7 proteins of indica and japonica rice were employed for generation of identity matrix using online tool SIAS (Sequence Identity And Similarity; http://imed.med.ucm.es/Tools/sias.html). Highlighted yellow color indicates corresponding indica and japonica OsTET proteins identity. OsTET indica and japonica amino acids share an average of 99% identity.

OsTET indica and japonica proteins identity matrix

**OsITET8.2**	100															
**OsTET8.3**	100	100														
**OsITET9**	38.23	38.23	100													
**OsITET10**	39.03	39.03	40.14	100												
**OsITET11**	35.53	35.53	37.50	58.36	100											
**OsITET12**	42.59	42.59	39.33	58.36	54.21	100										
**OsITET13**	38.40	38.40	38.23	63.19	50.54	63.76	100									
**OsITET14**	31.85	31.85	31.11	37.54	34.07	37.03	37.40	100								
**OsJTET8.2**	100	100	38.23	39.03	35.53	42.59	38.40	31.85	100							
**OsJTET8.3**	98.91	98.91	38.23	39.03	35.53	42.59	38.40	31.85	98.91	100						
**OsJTET9**	37.72	37.72	99.63	40.52	37.72	39.56	38.09	31.11	37.72	38.09	100					
**OsJTET10**	38.88	38.88	40	100	58.14	58.14	62.96	37.40	38.88	38.88	40.37	100				
**OsJTET11**	35.40	35.40	36.76	58.36	98.53	54.74	50.36	34.44	35.40	35.40	36.99	58.51	100			
**OsJTET12**	42.44	42.44	39.33	57.99	54.21	99.27	63.40	37.03	42.44	42.59	39.56	57.77	54.74	100		
**OsJTET13**	38.26	38.26	38.23	63.19	50.54	63.53	100	37.40	38.26	38.26	38.09	62.96	50.36	63.53	100	
**OsJTET14**	31.73	31.73	30.99	37.54	33.94	36.90	37.26	100	31.73	31.73	30.99	37.40	34.31	36.90	37.26	100
	**OsITET8.2**	**OsTET8.3**	**OsITET9**	**OsITET10**	**OsITET11**	**OsITET12**	**OsITET13**	**OsITET14**	**OsJTET8.2**	**OsJTET8.3**	**OsJTET9**	**OsJTET10**	**OsJTET11**	**OsJTET12**	**OsJTET13**	**OsJTET14**

Supplementary Figure 2 (B). Rice indica and japonica tetraspanin proteins identity matrix. OsTET8-14 proteins of indica and japonica rice were employed for generation of identity matrix using online tool SIAS (Sequence Identity And Similarity; http://imed.med.ucm.es/Tools/sias.html). Highlighted yellow color indicates corresponding indica and japonica OsTET proteins identity. OsTET indica and japonica amino acids share an average of 99% identity. Protein sequence of OsTET15 was not found in indica rice dataset and was therefore not included in the analysis.

Supplementary Figure 3

A

OsTET proteins identity matrix

OsTET1																	
OsTET2	33																
OsTET3	31.5	28.8															
OsTET4	36.9	46	30.2														
OsTET5	22.3	23.3	23.4	23.4													
OsTET6	25.6	25.5	23.8	24.4	44.4												
OsTET7	36.5	33.5	33.3	36.6	31	38.7											
OsTET8.1	39	27.9	22.9	25.7	15.5	17.6	23.8										
OsTET8.2	51.9	35.5	29.9	33.7	21.6	25.4	37.2	59.5									
OsTET8.3	53.7	35.5	30.8	33.7	21.6	25.4	37.2	59.5	98.9								
OsTET9	35.3	71.4	30.6	45.9	24.2	25.1	35.7	25.3	35	35							
OsTET10	39	38	36.5	35	25	31.3	46	26.2	37.6	37.6	40						
OsTET11	38.2	35.5	36	38.8	25	29.6	39.1	24	34.4	34.4	35.2	56					
OsTET12	40.6	38.3	36.1	36	25.8	30.2	43	29	40.7	40.7	39.2	56.3	52.7				
OsTET13	35.4	37.1	35.3	35.3	26.1	29.9	41.7	27.3	37.3	37.3	36.1	61.2	49.6	61.7			
OsTET14	32.4	30.3	27	28.2	20.1	21.7	30.2	21.5	31.8	31.8	30.9	36.8	33	35.8	36.7		
OsTET15	22.1	24.2	23.2	23.4	50	49.8	34.7	15.3	21.9	21.9	23.7	30.1	27.9	27.7	27.7	21.1	
	OsTET1	OsTET2	OsTET3	OsTET4	OsTET5	OsTET6	OsTET7	OsTET8.1	OsTET8.2	OsTET8.3	OsTET9	OsTET10	OsTET11	OsTET12	OsTET13	OsTET14	OsTET15

B

OsTET proteins similarity matrix

OsTET1																	
OsTET2	58.29																
OsTET3	42.82	40.13															
OsTET4	60.98	73.99	41.7														
OsTET5	46.18	55.6	32.51	56.95													
OsTET6	44.61	51.56	30.71	52.01	67.93												
OsTET7	61.43	65.02	44.84	64.57	54.93	55.38											
OsTET8.1	64.12	53.81	32.95	53.81	42.82	39.68	54.7										
OsTET8.2	78.02	61.88	41.7	62.55	48.87	46.18	64.12	76.23									
OsTET8.3	77.35	64.12	41.7	64.79	51.12	48.43	65.91	77.13	97.3								
OsTET9	58.07	90.58	41.25	75.33	55.6	51.12	65.69	52.91	61.21	63.45							
OsTET10	62.55	67.93	47.53	68.6	54.93	53.36	69.73	54.7	64.57	66.81	66.81						
OsTET11	60.76	65.69	47.53	67.26	51.56	50.44	65.47	52.24	62.1	64.34	65.47	78.69					
OsTET12	64.34	67.48	47.98	69.05	52.24	51.34	68.6	56.95	67.48	69.73	67.26	78.69	76.9				
OsTET13	63	66.81	46.86	68.38	53.81	50.44	67.93	54.26	64.79	67.04	65.69	82.51	74.66	82.73			
OsTET14	60.53	67.48	43.04	65.47	55.6	50	63.9	54.7	64.12	66.36	66.59	69.73	67.48	68.83	68.6		
OsTET15	44.61	53.13	32.06	52.91	72.64	71.52	54.03	40.8	46.18	48.43	52.91	52.24	50.44	51.34	50.67	51.34	
	OsTET1	OsTET2	OsTET3	OsTET4	OsTET5	OsTET6	OsTET7	OsTET8.1	OsTET8.2	OsTET8.3	OsTET9	OsTET10	OsTET11	OsTET12	OsTET13	OsTET14	OsTET15

Supplementary Figure 3. Rice tetraspanin proteins identity and similarity matrix. OsTET proteins identity (**A**) and similarity (**B**) matrix was generated by using online tool SIAS (Sequence Identity And Similarity; http://imed.med.ucm.es/Tools/sias.html). OsTET amino acids share an average of 34% identity and 48% similarity.

Supplementary Figure 4 (A)

A

OsTET EC1 domain identity matrix

**OsTET1**															
**OsTET2**	42.9														
**OsTET3**	38.1	52.4													
**OsTET4**	42.9	33.3	42.9												
**OsTET5**	28.6	28.6	33.3	38.1											
**OsTET6**	38.1	38.1	38.1	38.1	61.9										
**OsTET7**	42.9	38.1	38.1	52.4	38.1	42.9									
**OsTET8.2**	52.4	33.3	38.1	42.9	38.1	38.1	42.9								
**OsTET8.3**	52.4	33.3	38.1	42.9	38.1	38.1	42.9	100.0							
**OsTET9**	42.9	81.0	42.9	28.6	28.6	38.1	38.1	33.3	33.3						
**OsTET10**	42.9	52.4	38.1	28.6	33.3	47.6	57.1	38.1	38.1	52.4					
**OsTET11**	19.0	23.8	23.8	19.0	14.3	19.0	38.1	19.0	19.0	19.0	28.6				
**OsTET12**	33.3	38.1	38.1	38.1	38.1	33.3	52.4	38.1	38.1	33.3	33.3	42.9			
**OsTET13**	23.8	42.9	38.1	33.3	38.1	38.1	57.1	28.6	28.6	33.3	47.6	42.9	71.4		
**OsTET14**	38.1	47.6	52.4	38.1	47.6	47.6	42.9	38.1	38.1	47.6	42.9	23.8	47.6	47.6	
**OsTET15**	33.3	28.6	33.3	38.1	57.1	61.9	38.1	33.3	33.3	28.6	28.6	19.0	33.3	33.3	38.1
	**OsTET1**	**OsTET2**	**OsTET3**	**OsTET4**	**OsTET5**	**OsTET6**	**OsTET7**	**OsTET8.2**	**OsTET8.3**	**OsTET9**	**OsTET10**	**OsTET11**	**OsTET12**	**OsTET13**	**OsTET14**	**OsTET15**

OsTET EC1 domain similarity matrix

**OsTET1**															
**OsTET2**	42.9														
**OsTET3**	47.6	52.4													
**OsTET4**	42.9	38.1	47.6												
**OsTET5**	28.6	33.3	33.3	47.6											
**OsTET6**	42.9	42.9	42.9	42.9	61.9										
**OsTET7**	52.4	47.6	57.1	61.9	38.1	42.9									
**OsTET8.2**	71.4	38.1	52.4	57.1	47.6	52.4	61.9								
**OsTET8.3**	71.4	38.1	52.4	57.1	47.6	52.4	61.9	100.0							
**OsTET9**	42.9	95.2	57.1	38.1	33.3	42.9	42.9	38.1	38.1						
**OsTET10**	57.1	57.1	52.4	33.3	33.3	47.6	61.9	52.4	52.4	52.4					
**OsTET11**	28.6	23.8	33.3	23.8	19.0	19.0	42.9	28.6	28.6	23.8	33.3				
**OsTET12**	38.1	38.1	42.9	42.9	38.1	38.1	61.9	47.6	47.6	38.1	42.9	47.6			
**OsTET13**	33.3	42.9	42.9	42.9	38.1	38.1	71.4	42.9	42.9	38.1	57.1	47.6	76.2		
**OsTET14**	42.9	57.1	52.4	42.9	47.6	47.6	47.6	38.1	38.1	61.9	47.6	23.8	47.6	47.6	
**OsTET15**	33.3	33.3	33.3	38.1	57.1	61.9	42.9	38.1	38.1	33.3	28.6	19.0	42.9	38.1	38.1
	**OsTET1**	**OsTET2**	**OsTET3**	**OsTET4**	**OsTET5**	**OsTET6**	**OsTET7**	**OsTET8.2**	**OsTET8.3**	**OsTET9**	**OsTET10**	**OsTET11**	**OsTET12**	**OsTET13**	**OsTET14**	**OsTET15**

Supplementary Figure 4 (A). Identity and similarity matrix of EC1 domain in rice tetraspanin proteins. The data was generated by using online tool SIAS (Sequence Identity And Similarity; http://imed.med.ucm.es/Tools/sias.html). EC1 domain amino acids share an average of 22.4% identity and 30.2% similarity.

B **Supplementary Figure 4 (B)**

OsTET EC2 domain identity matrix

	OsTET1	OsTET2	OsTET3	OsTET4	OsTET5	OsTET6	OsTET7	OsTET8.2	OsTET8.3	OsTET9	OsTET10	OsTET11	OsTET12	OsTET13	OsTET14	OsTET15
OsTET1																
OsTET2	50.8															
OsTET3	41.2	41.7														
OsTET4	51.3	62.0	40.6													
OsTET5	29.9	35.3	25.7	32.6												
OsTET6	42.8	46.0	34.8	42.8	59.9											
OsTET7	46.5	48.7	38.5	45.5	45.5	61.5										
OsTET8.2	73.8	53.5	39.6	48.7	28.9	40.6	45.5									
OsTET8.3	73.8	53.5	39.6	48.7	28.9	40.6	45.5	100								
OsTET9	51.3	77.5	44.9	62.6	34.8	44.4	47.6	50.3	50.3							
OsTET10	55.6	56.7	47.1	52.9	35.3	48.7	56.1	54.0	54.0	57.2						
OsTET11	55.6	56.1	49.7	56.1	35.3	46.5	51.9	54.5	54.5	55.6	73.3					
OsTET12	57.2	54.0	49.2	52.4	34.8	43.9	48.1	54.0	54.0	55.1	71.7	69.5				
OsTET13	52.4	53.5	46.0	49.7	32.6	43.9	46.5	52.9	52.9	52.9	71.1	65.8	72.2			
OsTET14	52.4	49.2	40.6	46.0	31.0	40.6	44.9	52.4	52.4	50.3	54.0	52.4	52.9	54.0		
OsTET15	35.8	40.1	31.0	36.4	66.3	67.9	56.7	33.7	33.7	39.0	43.3	41.2	39.0	38.0	36.9	

OsTET EC2 domain similarity matrix

	OsTET1	OsTET2	OsTET3	OsTET4	OsTET5	OsTET6	OsTET7	OsTET8.2	OsTET8.3	OsTET9	OsTET10	OsTET11	OsTET12	OsTET13	OsTET14	OsTET15
OsTET1																
OsTET2	58.8															
OsTET3	49.2	51.9														
OsTET4	58.8	69.0	49.7													
OsTET5	39.0	39.0	31.6	41.7												
OsTET6	50.3	51.9	40.6	50.3	64.7											
OsTET7	55.1	57.2	46.0	54.0	50.3	67.4										
OsTET8.2	84.0	60.4	49.7	57.8	39.0	48.7	54.5									
OsTET8.3	84.0	60.4	49.7	57.8	39.0	48.7	54.5	100								
OsTET9	57.8	85.6	51.9	70.6	41.2	50.8	54.0	59.4	59.4							
OsTET10	65.8	67.9	57.2	65.2	41.7	54.0	63.1	64.7	64.7	65.8						
OsTET11	66.8	65.8	59.9	67.4	40.6	52.4	58.3	65.2	65.2	64.2	78.1					
OsTET12	66.3	63.6	58.8	65.2	41.7	51.3	55.1	67.9	67.9	63.6	76.5	75.9				
OsTET13	64.2	63.6	57.8	61.5	39.6	49.7	55.1	63.6	63.6	61.5	77.5	74.3	79.7			
OsTET14	61.0	63.1	52.4	57.2	40.6	50.3	52.9	63.1	63.1	59.9	65.8	67.4	65.8	63.6		
OsTET15	44.4	47.6	38.5	46.0	72.2	73.3	60.4	43.9	43.9	46.5	48.1	46.5	46.5	46.0	44.4	

Supplementary Figure 4 (B). Identity and similarity matrix of EC2 domain in rice tetraspanin proteins. The data was generated by using online tool SIAS (Sequence Identity And Similarity; http://imed.med.ucm.es/Tools/sias.html). EC2 domain amino acids share an average of 37.6% identity and 41.6% similarity.

Supplementary Figure 5

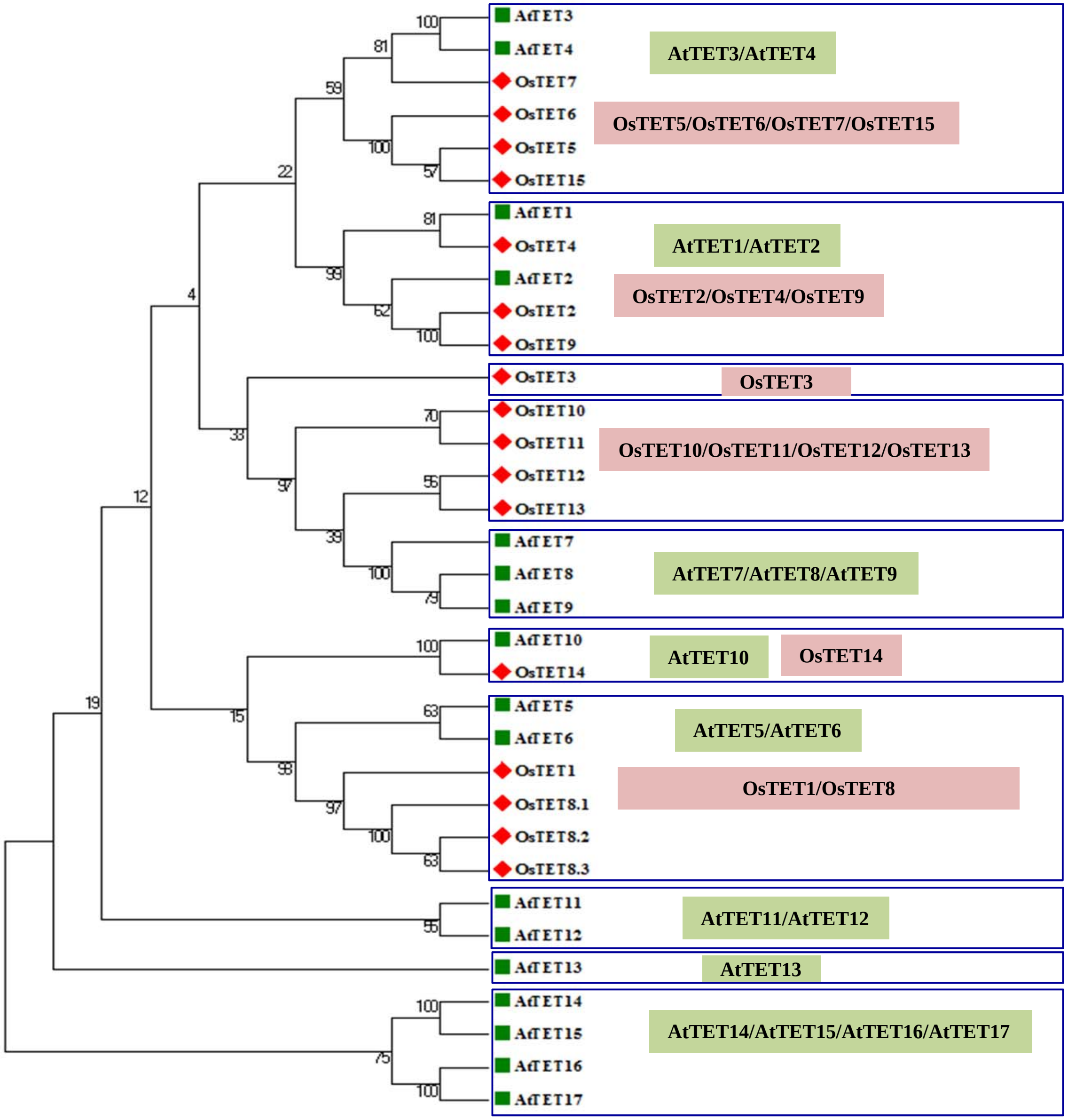

Supplementary Figure 5. Phylogenetic tree of *Arabidopsis* and rice tetraspanin proteins. An un-rooted neighbor-joining method was employed to construct phylogenetic tree for full-length protein sequences of *Arabidopsis* and rice tetraspanins. Multiple sequence alignment was carried out using ClustalX 2.1 and phylogenetic tree was generated using MEGA6. Number indicated above each branch represents bootstrap percentage value. Boxes represent clustering of TET proteins in clades based on bootstrap values ≥ 50%.

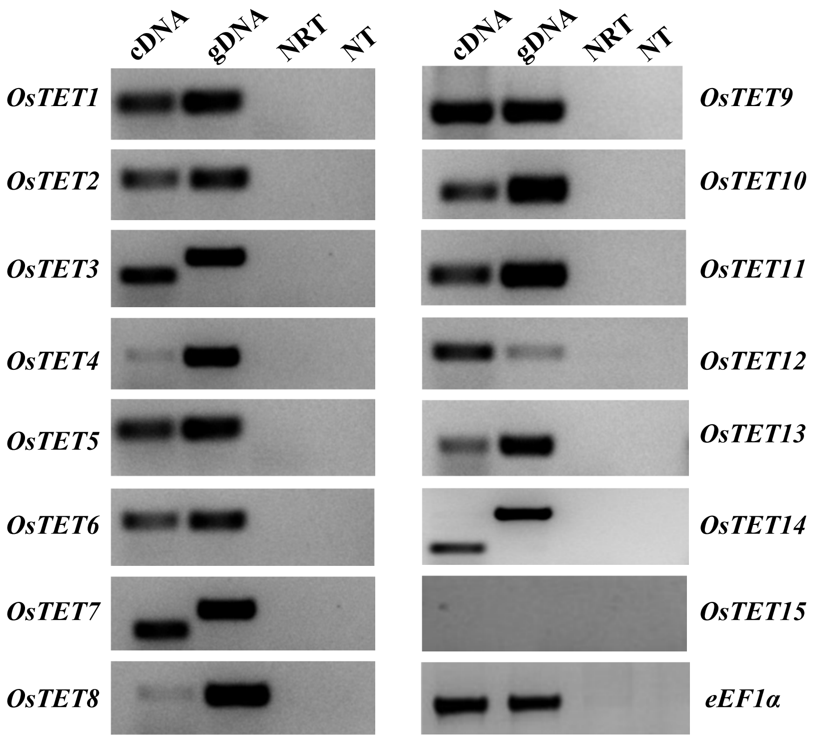

Supplementary Figure 6. Testing of primers designed for quantitative PCR of *OsTET* genes. qPCR primers were used for amplification of a portion of *OsTET*s from seven-day old seedling cDNA, genomic DNA (gDNA), NRT: no reverse transcription control, and NT: no template control. Rice eukaryotic translation elongation factor 1alpha (*eEF1α*) was used as an internal control. All *OsTET*s could be amplified, except *OsTET15*.

A

***OsTET15*-FP2**

ATGGCGTTATTTGGGGTAAGAGAGATGAGACGATTTGAGAGAATGACGCCGGAAAGGCGAGCTGAATATCTACTAGGCATGGCCGGTGTCGTGACGTTGGTAACGTCGATCCTCCTCCTGTTAAGC

***OsTET15*-RP2**

GGCAGCACCTATGGGTACGGCCCTAAGGTTTGCACCGGCCGTGGCGTCTTCTTTAGCCCCACGATCGCTCTCGGCCTCCTGCTGATGGCCGCCTTCATTTTGGGTACGTGCGGCCAGAGGTACGGTG

***OsTET15*-FP4**

ATGAGTGCCTCTTTGGTTGCTACCTCCTCGGTCTCCTCATCGCCTTCCCCCTCCTCCTTGCCTTCATAATCTTCGGCTACGTCGCTGTTGGAGGCATCGACCTCGGCGGCGTAAGTATTCGCGAGTACA

***OsTET15*-RP4** ***OsTET15*-FP5**

ATCTGGAGGAGTACAGCGGGTGGCTCAGGGGCCGTGTGGCCGACCCGCACTACTGGGAAACAACCAGCGCATGCCTACGCGACGGGAACGTCTGCAGCGGCATGACGCGGTTGGTACGCGACCCA

***OsTET15*-RP5**

AACACCGGCATATTCGTCCCTGAGTTATCCCGGTATGAAAGGTGGCTCAAGGAACATGGGATAAAAAAAGGCGTGCACGTAATGTCGCCTATCGAGgtatgtacgtagcagctagcttcgatataattgatggatgatatc
cccgagcccaagctcaggtgtaacttcggtattagcctatgtatgtccaaccttagtgtacaaaacttgatttattttgaaagggagagggtagtatcttctttgacgtatgggccggtccacctttgaaaccacatgaatgcaaccgcgtgcgtggtgacggtttttttgtttaggtccgacaaagat
gaccaccatcgatgacggctctaacacatatgagtttccacatcgctgaccactaaaccgctaccagaccgtctgacatagatgagggttcccgccagacagaaatgacttgattcgcactagcgcactcgggatgaagcggtgcaccactttgttttagggagttcaaaagacttccagctt
agtatacgtacgttgtgctaggttaattatagcttgcatttcattttttttctttcctttcctattccctgctttattttttcagtgatgatgatatattttgttcaatacttctttatttatgagcaattctacggttcttaggaaggtaccatgagatagcaaaatttacacttaaatttcggtacctcacagtacctag
ataccatgagttaccaaatttacaatagaaaaaatggtatttcatggtatctcctcaaggactttaaaattgctctttatttattcatttcgatatttcacttAattggaacctcgccttataactgaatatatatatgctgcacttggcagTCTGGATGCTGCAAGCCACCGACATCAT

***OsTET15*-FP1** ***OsTET15*-FP3**

GTGGTTTCAGATATGTCAACGGGACGACGTGGATACCAACACCAGCAGCAGCAGGCACCCCTGCCGCGGCGACCAACGTCGACTGCAGCAGGTGGAGCAACAACCAACAGACGCTCTGCTTCCAG

***OsTET15*-RP1** ***OsTET15*-RP3**

TGCGACTCATGCAAGGCTGGCTTCCTGGACGACATCAAGAAAGCCTGGAGTTTCGATGCCCTGTATCCAATTCTCGCGTTGGTCGGAGCCTTTCTTTCATGCTTTGCAGGCGTCAAGTATTGGAGACC
AAGACTAGACACTGGGTACTCACTAATTAGAGAACGAGCAGTGGCTTAA

B

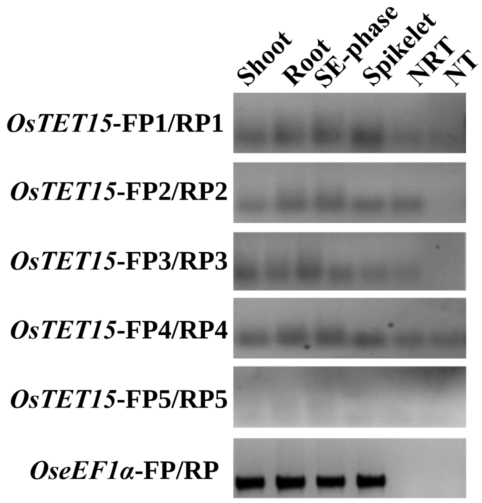

Supplementary Figure 7. (A) Details of different combinations of quantitative PCR primers designed for amplification of *OsTET15*. The binding sites of qPCR primers are highlighted on *OsTET15* genomic sequence. **(B) Testing of various combinations of primers designed for amplification of *OsTET15*.** Amplification of *OsTET15* using cDNAs prepared from different tissues (shoot, root, stem elongation phase or SE phase, spikelet) with different primer combinations. *OsTET15* could not be detected in various tissues included in our study. Rice eukaryotic translation elongation factor 1alpha (*eEF1α*) was used as an internal control. NRT: no reverse transcription control, NT: no template control.

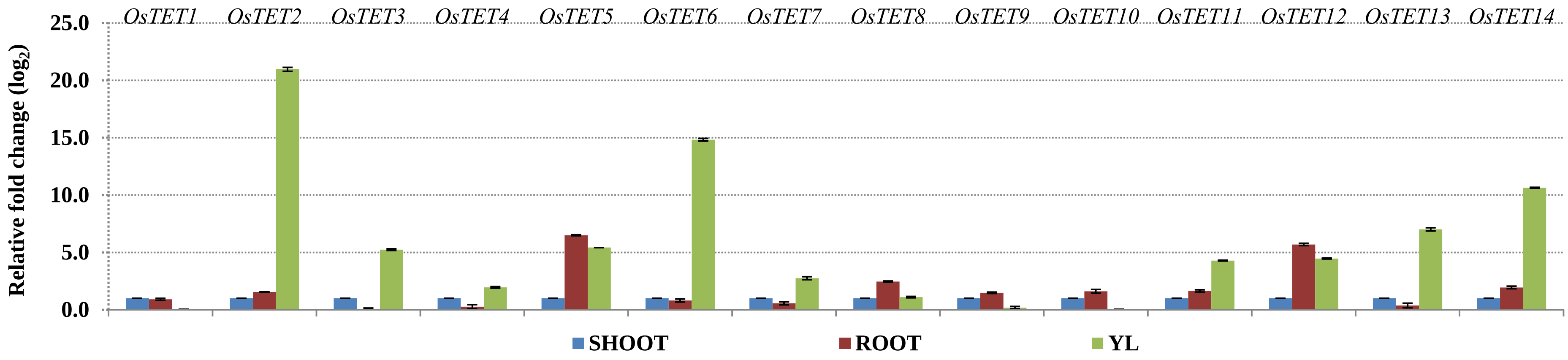

Supplementary Figure 8. Expression profile of tetraspanin genes in different tissues of rice. Quantitative PCR analysis of *OsTET* transcript levels in various tissues: shoot, root and young leaf (YL) was performed. Normalized fold change ($\log_2$ scale) was calculated relative to that in shoot tissue. For normalization *eEF-1α* was used as an internal control. Three biological replicates and two technical replicates were included in the study. Error bars represent standard error (SE) of three independent biological replicates.

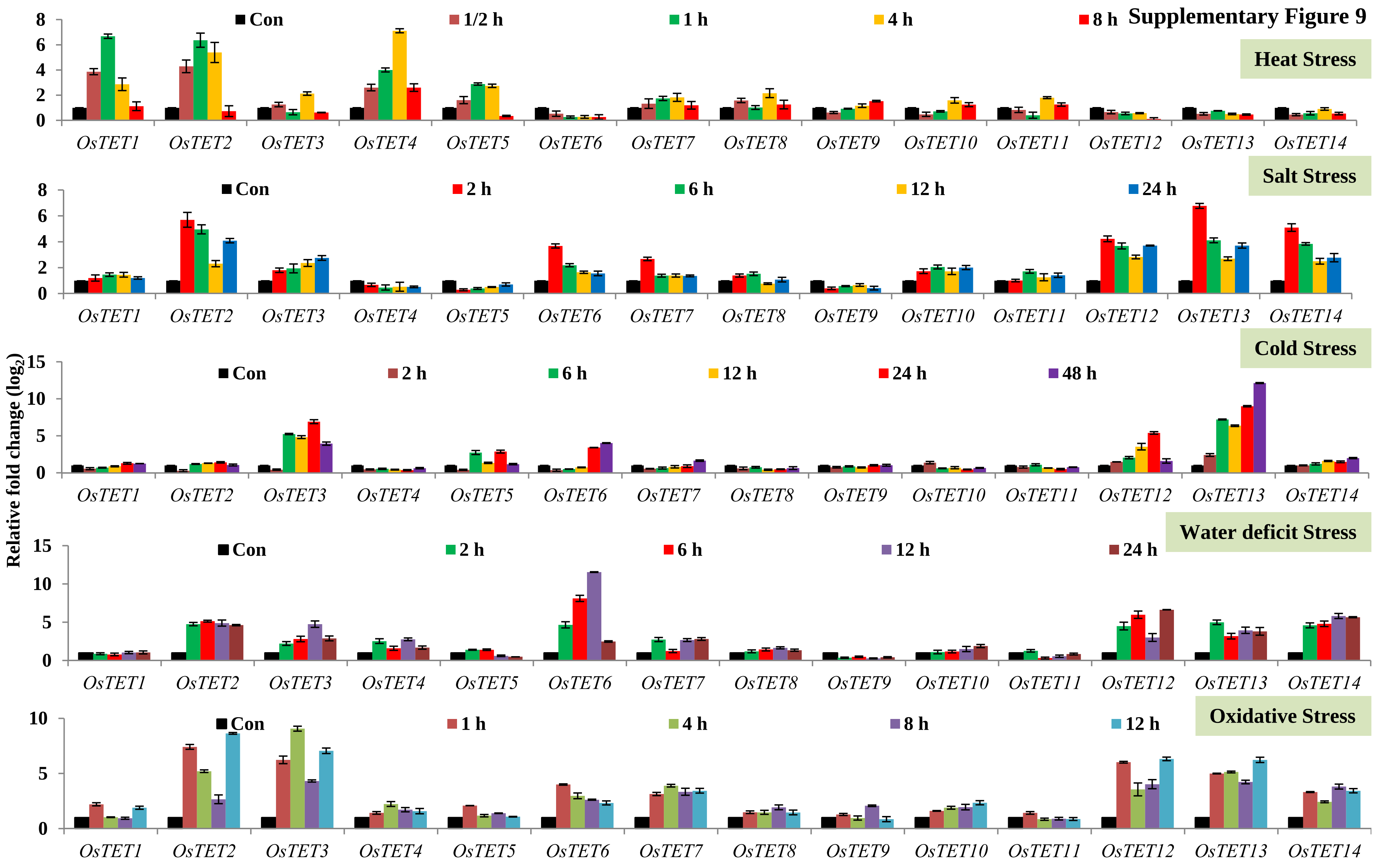

Supplementary Figure 9. Expression profiling of tetraspanin genes under different abiotic stresses. Seven-day-old seedlings were exposed to different abiotic stresses such as heat stress (HS) at 42°C; salinity stress (SS) with 200 mM NaCl; water deficit stress (WDS) imposed by 15% PEG; cold stress (CS) at 4°C; oxidative stress (OS) with 10 mM H_2O_2 for different time durations (in h). Normalized log(2)-fold change values were calculated relative to the expression in control unstressed sample. For normalization *eEF-1α* was used as an internal control. Three biological replicates for each treatment and two technical replicates were included in this study. Error bars represent standard error (SE) of three independent biological replicates.

Supplementary Figure 10

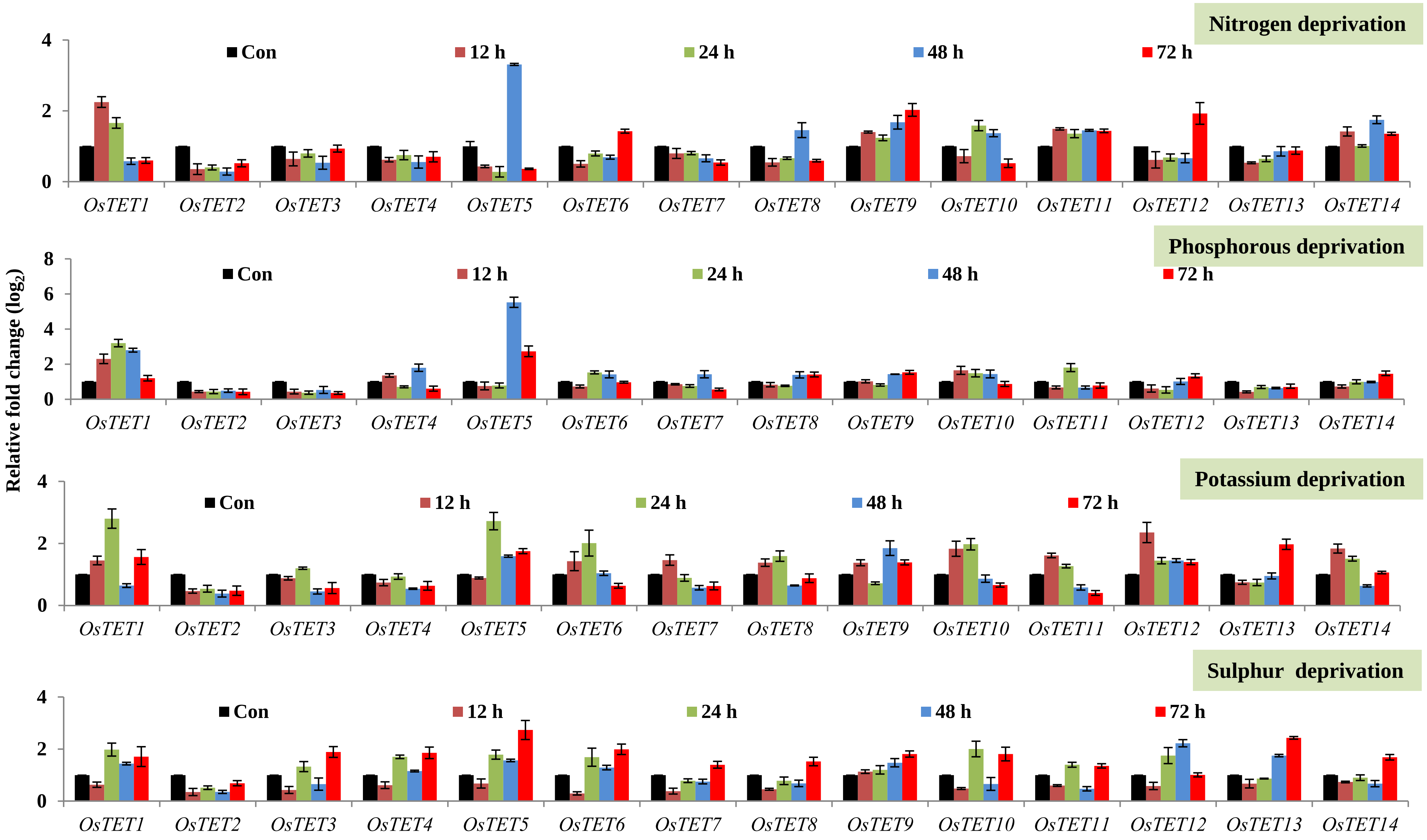

Supplementary Figure 10. Expression profile of rice tetraspanin genes during nutrient deprivation conditions. Seven-day-old rice seedlings were grown in different nutrient deprivation media such as nitrogen deprivation (N), phosphorous deprivation (P), potassium deprivation (K), sulphur deprivation (S) for different time durations (in h). Expression levels of tetraspanin genes were determined by quantitative PCR and normalized fold change (log_2 scale) was calculated relative to that in unstressed seedlings. For normalization *eEF-1α* was used as an internal control. Three biological replicates and two technical replicates were included in the study. Error bars represent standard error (SE) of three independent biological replicates.

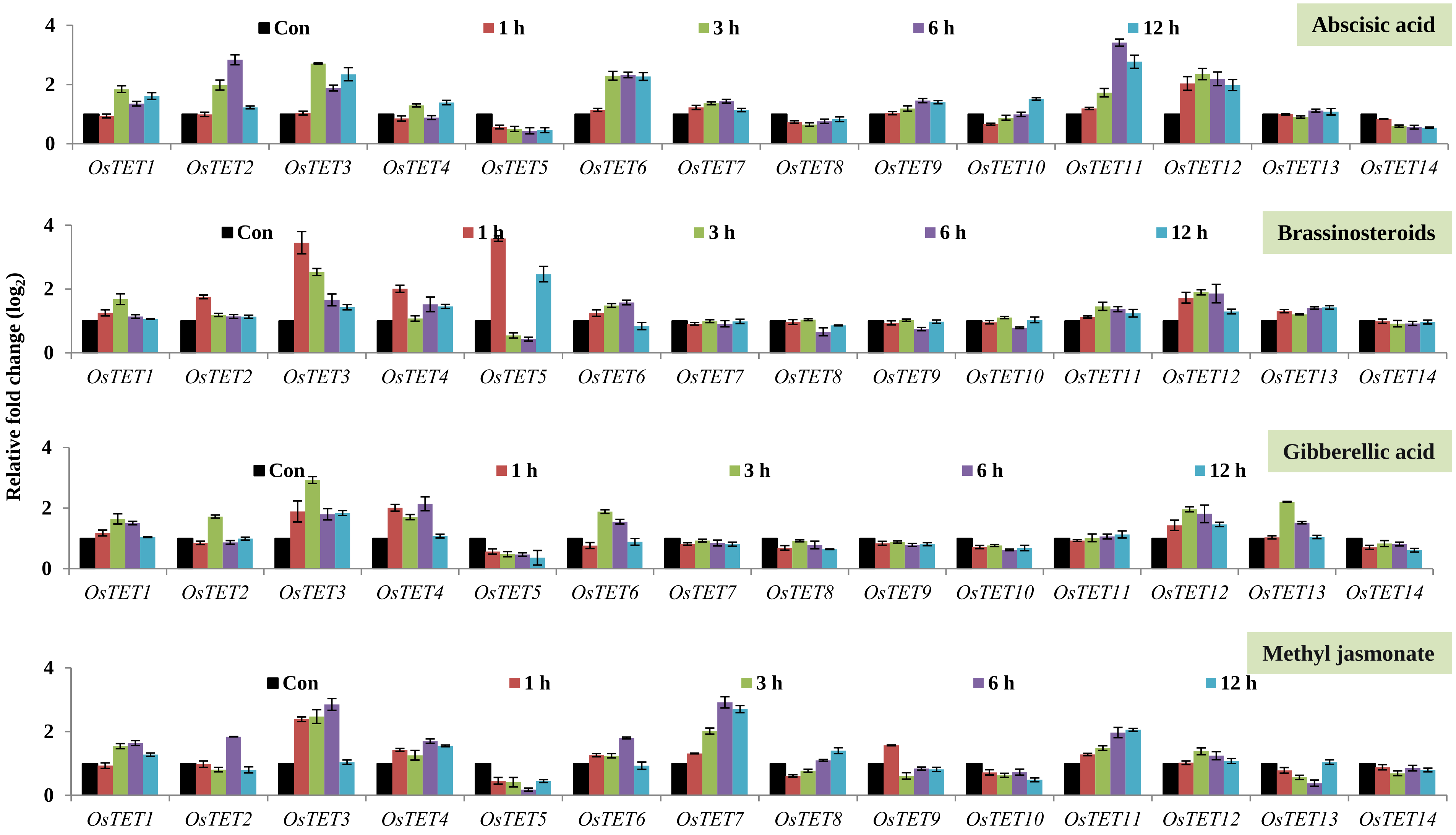

Supplementary Figure 11. Expression profile of tetraspanin genes in rice seedlings exposed to different hormones. Seven-day-old seedlings were exposed to exogenous hormones, namely abscisic acid (ABA), brassinosteroids (BS), gibberellic acid (GA), and methyl jasmonate (MeJA) for different time durations (in h). Expression levels of tetraspanin genes were determined by quantitative PCR and normalized fold change ($\log_2$ scale) was calculated relative to that in untreated seedlings. For normalization *eEF-1α* was used as an internal control. Three biological replicates and two technical replicates were included in the study. Error bars represent standard error (SE) of three independent biological replicates.

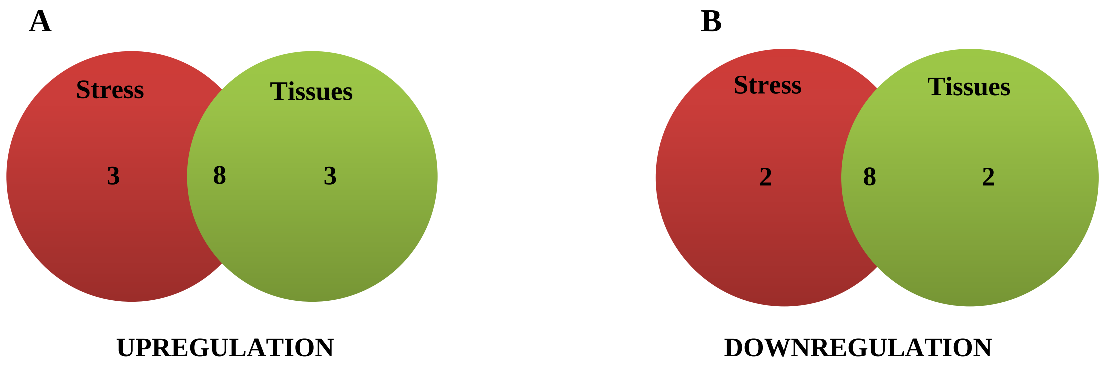

Supplementary Figure 12. Overlap in expression pattern of rice tetraspanin genes in different tissues and abiotic stresses. Venn diagram representing overlap of OsTET genes exhibiting significant change (≥2 fold change in log2 scale) in expression between tissues and abiotic stresses. Both upregulated **(A)** and downregulated **(B)** genes are shown. Eleven tissues: shoot, root, YL, AT-phase, SE-phase, spikelets, YFL, MFL and S1, S2, S3 stages of flag leaf senescence and five abiotic stresses: heat stress (HS), salinity stress (SS), cold stress (CS), water deficit stress (WDS) and oxidative stress (OS) were included in this study

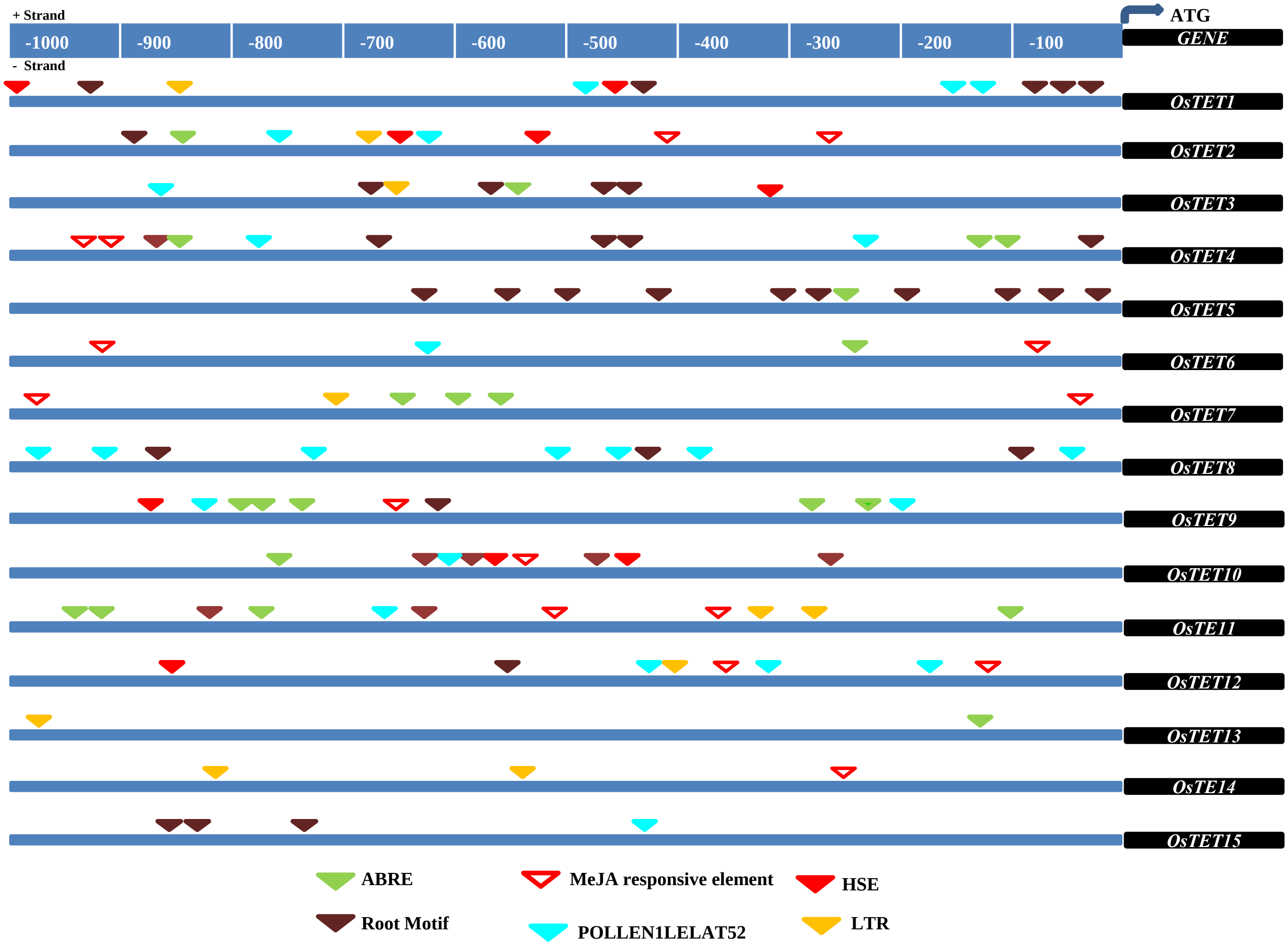

Supplementary Figure 13. ***In silico*** **analysis of rice tetraspanin promoter for identification of cis-regulatory elements**. Nucleotide sequences 1 kb upstream of translational start site of 15 tetraspanin genes were analyzed using New PLACE and PlantCARE databases. Different classes of *cis*-regulatory elements are indicated by triangle shapes of different colors. A scale is shown on the top to indicate specific location of these elements.

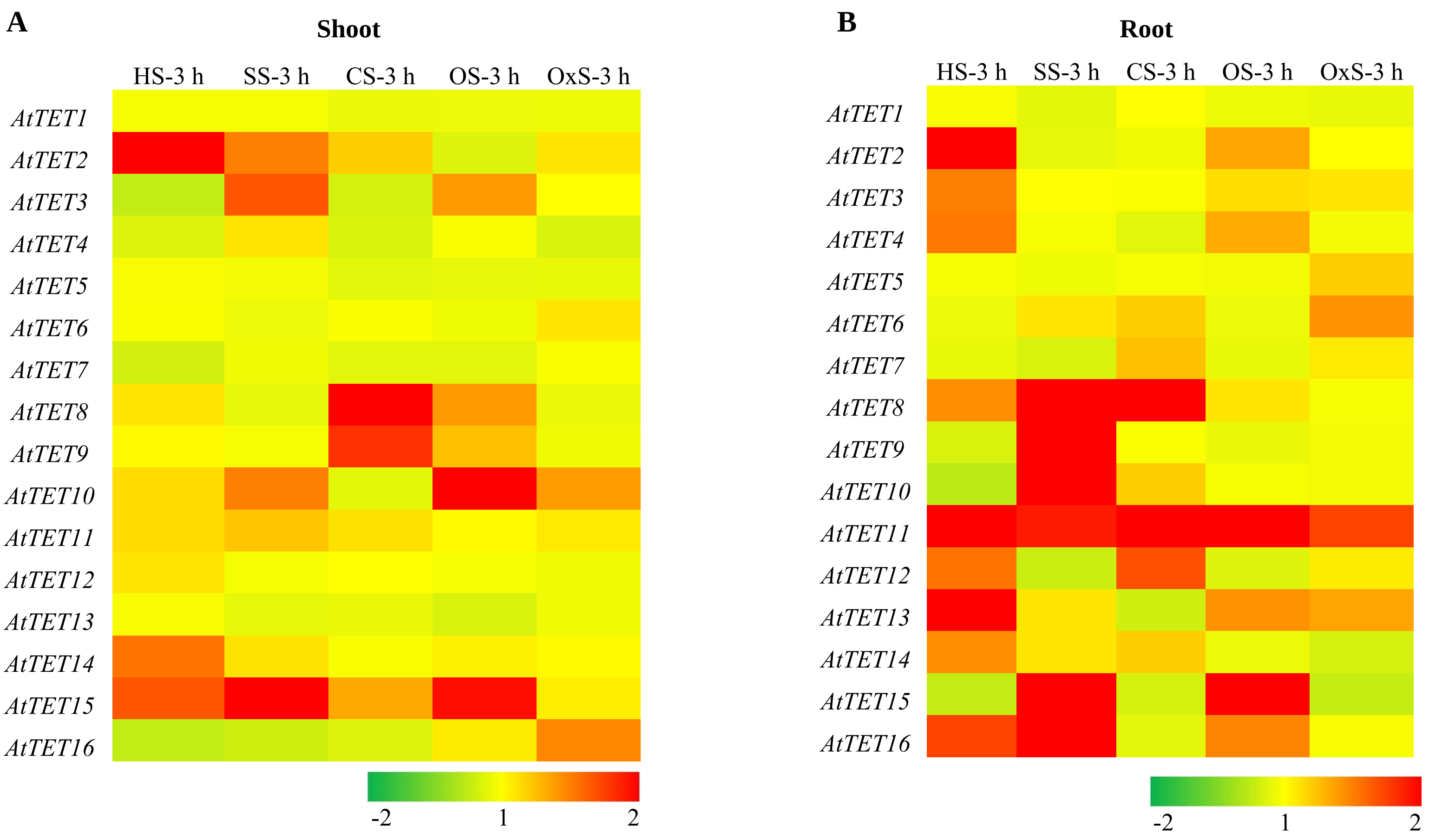

Supplementary Figure 14. Heat map representing the expression pattern of *Arabidopsis* tetraspanin genes under various abiotic stresses in shoots (A) and roots (B) constructed on the basis of publicly available expression database Arabidopsis eFP Browser (http://bar.utoronto.ca/efp/cgi-bin/efpWeb.cgi). HS: heat stress at 38ºC, CS: cold stress at 4±ºC, OS: osmotic stress by treating with 300 mM mannitol, SS: salinity stress by exposing to150 mM NaCl, OxS: oxidative stress by treatment with 10 µM methyl viologen. Eighteen-day-old Arabidopsis plants were employed for the experiment.

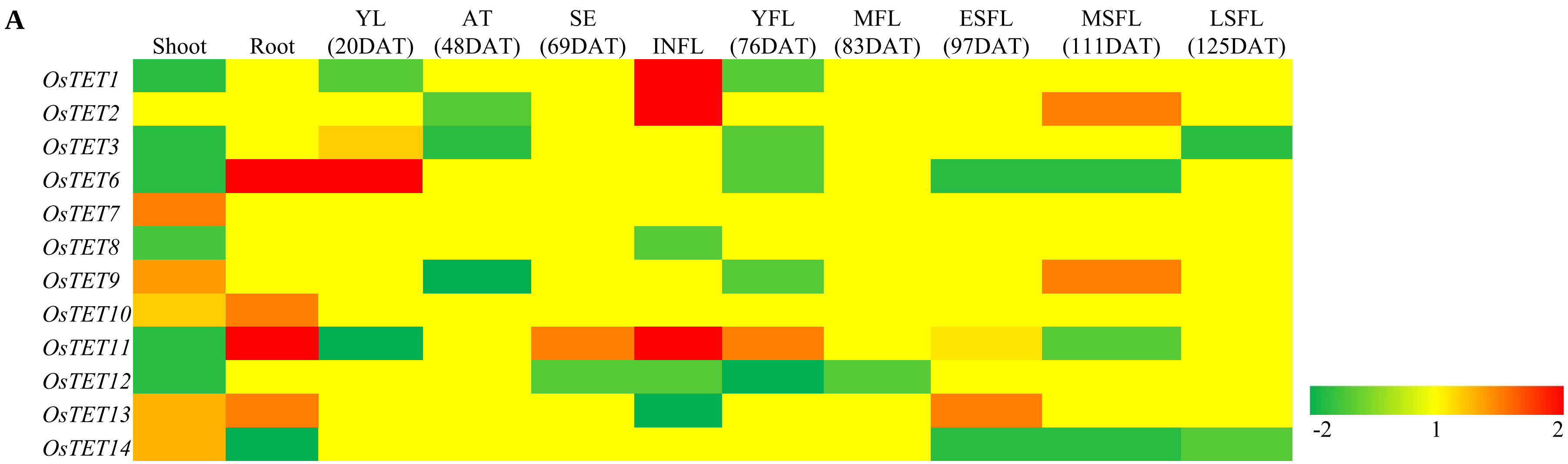

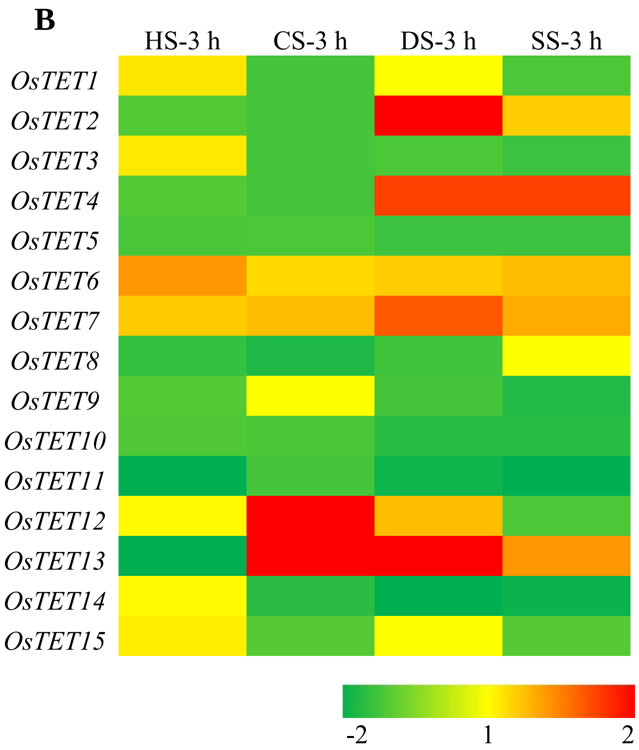

Supplementary Figure 15. Heat map representing the expression pattern of rice tetraspanin genes in different tissues (A) and abiotic stresses (B) constructed on the basis of publicly available expression databases, RiceXpro (http://ricexpro.dna.affrc.go.jp/index.html) and Genevestigator (https://genevestigator.com/gv/), respectively. The experiments were carried out in japonica rice variety, Nipponbare. When compared with the experiments conducted in this paper with indica rice variety (**please refer to Figures 3-6**) several of the genes exhibited similar pattern. *OsTET4*, *OsTET5* and *OsTET15* expression profile was not found in RiceXpro and was therefore not included in the analysis.DAT: days after transplant, YL: young leaf, AT: active tillering phase, SE: stem elongation phase, Inf: inflorescence, YFL: young flag leaf, MFL: mature flag leaf, ESFL: early senescence flag leaf , MSFL: mid senescence flag leaf, LSFL: late senescence flag leaf, HS: heat stress at 42ºC, CS: cold stress at 4±ºC, DS: desiccation stress by drying rice seedlings between folds of tissue paper at 28±1ºC, SS: salinity stress by 200mM NaCl. For abiotic stress experiments seven-day-old seedlings were employed. Fold change in expression levels is presented.
